# Supplementary material for: HELZ is a RNA-DNA helicase that resolves R loops to facilitate homologous recombination repair
Source: Nat Commun. 2026 Jul 23;17:6968. doi: 10.1038/s41467-026-75089-3 (PMC13396810; doi:10.1038/s41467-026-75089-3)
Supplement: Supplementary file 1 — Supplementary Information [file 41467_2026_75089_MOESM1_ESM.pdf]

Supplementary Materials for

HELZ is a RNA-DNA helicase that resolves R loops to facilitate homologous recombination repair

Authors: Ramona Haji-Seyed-Javadi[1], Allyson E. Koyen[1], Sandip K. Rath[1], Bo Wu[2], Matthew Z. Madden[1], Yingzi Hou[3], Priya Kapoor-Vazirani[1], Roshika Roshika[1], Meili Aiello[1], Nho Cong Luong[1], Fatmata Sesay[1], Wei-Che Tseng[1], John S. Kim[1], Tony Tan[1], Seohyun Kim[1], Boya Gao[4], Boying S. Song[1], Anna M. Kenney[5], Erin C. Connolly[1], Lily Yang[6], Blerta Xhemalce[7], Xiaoxian Li[8], Jeffrey M. Switchenko[9], Xiaofeng Yang[1], Zachary S. Buchwald[1], Xingming Deng[1], Kyle M. Miller[1], Bing Yao[3], Li Lan[4], Weixing Zhao[2], and David S. Yu[1*]

Corresponding author: dsyu@emory.edu

The PDF file includes:

Figs. S1 to S10

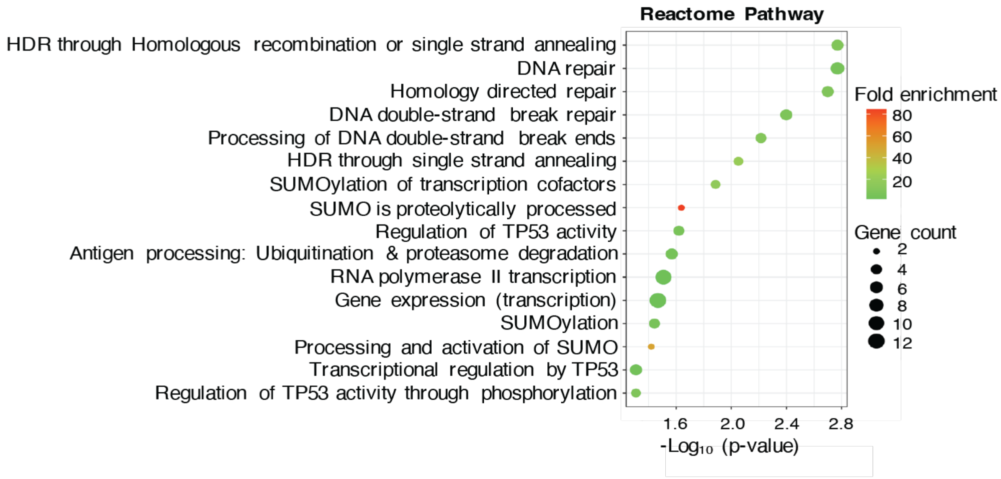

Reactome Pathway
HDR through Homologous recombination or single strand annealing
DNA repair
Homology directed repair
DNA double-strand break repair
Processing of DNA double-strand break ends
HDR through single strand annealing
SUMOylation of transcription cofactors
SUMO is proteolytically processed
Regulation of TP53 activity
Antigen processing: Ubiquitination & proteasome degradation
RNA polymerase II transcription
Gene expression (transcription)
SUMOylation
Processing and activation of SUMO
Transcriptional regulation by TP53
Regulation of TP53 activity through phosphorylation
1.6
2.0
2.4
2.8
-Log10 (p-value)
Fold enrichment
80
60
40
20
Gene count
2
4
6
8
10
12

Figure S1. Reactome pathway analysis. Reactome pathway analysis of the 62 etoposide sensitization hits highlights diverse roles in the DDR. The analysis was conducted using DAVID functional annotation bioinformatics tools. Significant pathways ($p < 0.05$) are displayed. Source data are provided as a Source Data file

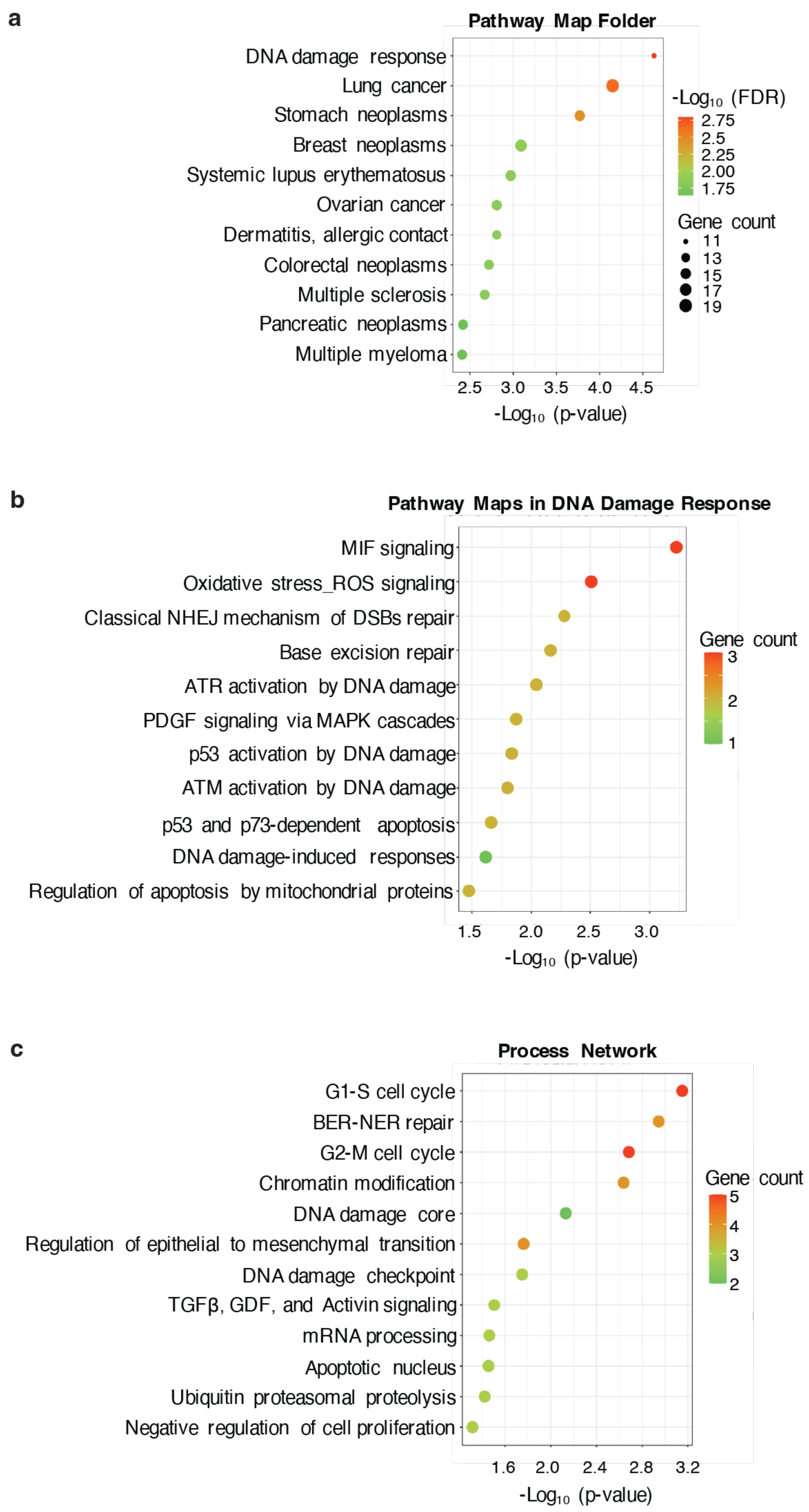
a
Pathway Map Folder
DNA damage response
Lung cancer
Stomach neoplasms
Breast neoplasms
Systemic lupus erythematosus
Ovarian cancer
Dermatitis, allergic contact
Colorectal neoplasms
Multiple sclerosis
Pancreatic neoplasms
Multiple myeloma
2.5 3.0 3.5 4.0 4.5
-Log10 (p-value)
-Log10 (FDR)
2.75 2.5 2.25 2.00 1.75
Gene count
11 13 15 17 19
b
Pathway Maps in DNA Damage Response
MIF signaling
Oxidative stress_ROS signaling
Classical NHEJ mechanism of DSBs repair
Base excision repair
ATR activation by DNA damage
PDGF signaling via MAPK cascades
p53 activation by DNA damage
ATM activation by DNA damage
p53 and p73-dependent apoptosis
DNA damage-induced responses
Regulation of apoptosis by mitochondrial proteins
1.5 2.0 2.5 3.0
-Log10 (p-value)
Gene count
3 2 1
c
Process Network
G1-S cell cycle
BER-NER repair
G2-M cell cycle
Chromatin modification
DNA damage core
Regulation of epithelial to mesenchymal transition
DNA damage checkpoint
TGFβ, GDF, and Activin signaling
mRNA processing
Apoptotic nucleus
Ubiquitin proteasomal proteolysis
Negative regulation of cell proliferation
1.6 2.0 2.4 2.8 3.2
-Log10 (p-value)
Gene count
5 4 3 2

Figure S2. MetaCore pathway map and process network enrichment analysis. MetaCore pathway map and process network enrichment analysis based on the 62 sensitization hits. **a** Pathway map folder. **b** Pathway maps focusing on the DNA damage response. **c** Process network.

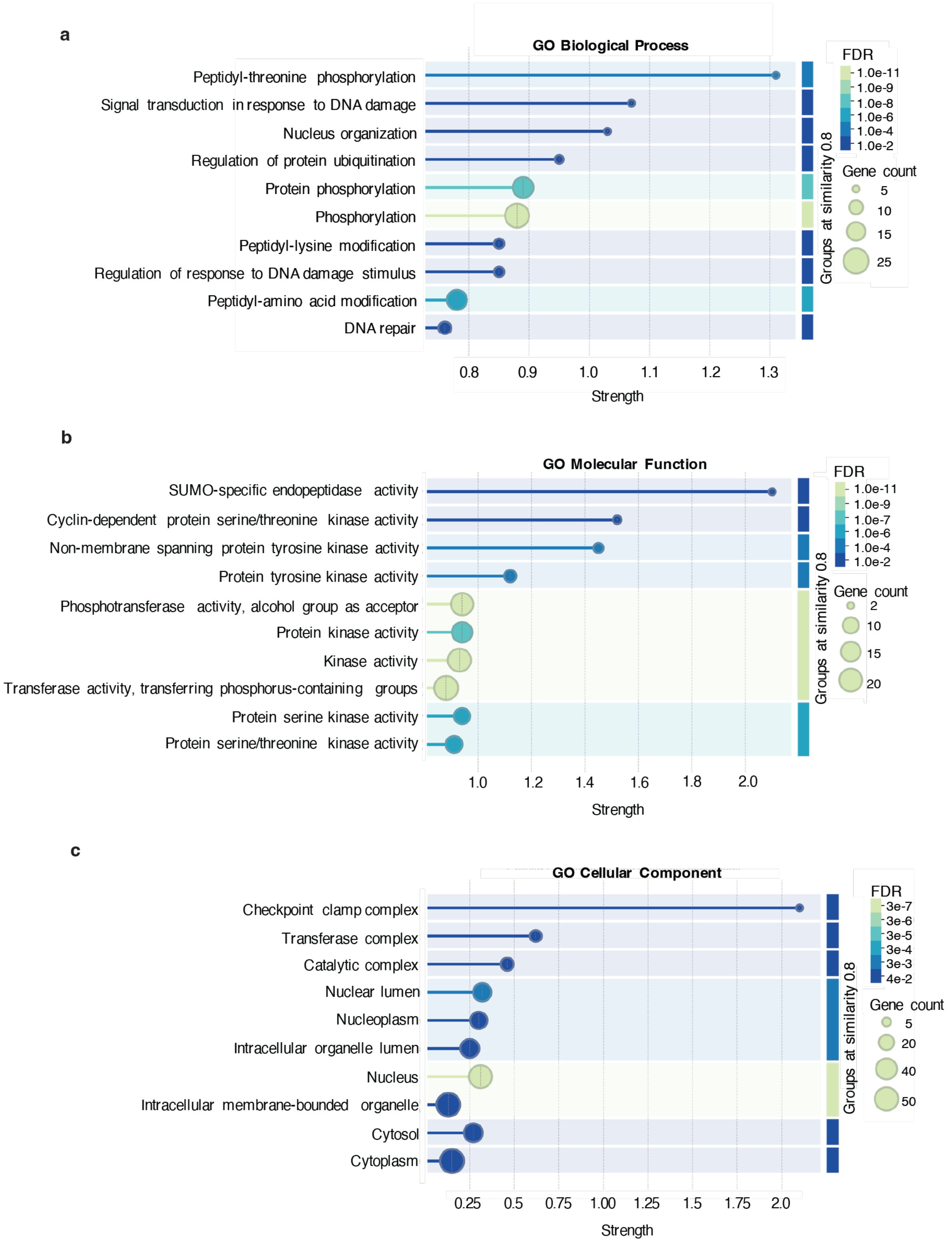
a
GO Biological Process
Peptidyl-threonine phosphorylation
Signal transduction in response to DNA damage
Nucleus organization
Regulation of protein ubiquitination
Protein phosphorylation
Phosphorylation
Peptidyl-lysine modification
Regulation of response to DNA damage stimulus
Peptidyl-amino acid modification
DNA repair
0.8 0.9 1.0 1.1 1.2 1.3
Strength
Groups at similarity 0.8
FDR
1.0e-11
1.0e-9
1.0e-8
1.0e-6
1.0e-4
1.0e-2
Gene count
5
10
15
25
b
GO Molecular Function
SUMO-specific endopeptidase activity
Cyclin-dependent protein serine/threonine kinase activity
Non-membrane spanning protein tyrosine kinase activity
Protein tyrosine kinase activity
Phosphotransferase activity, alcohol group as acceptor
Protein kinase activity
Kinase activity
Transferase activity, transferring phosphorus-containing groups
Protein serine kinase activity
Protein serine/threonine kinase activity
1.0 1.2 1.4 1.6 1.8 2.0
Strength
Groups at similarity 0.8
FDR
1.0e-11
1.0e-9
1.0e-7
1.0e-6
1.0e-4
1.0e-2
Gene count
2
10
15
20
c
GO Cellular Component
Checkpoint clamp complex
Transferase complex
Catalytic complex
Nuclear lumen
Nucleoplasm
Intracellular organelle lumen
Nucleus
Intracellular membrane-bounded organelle
Cytosol
Cytoplasm
0.25 0.5 0.75 1.00 1.25 1.5 1.75 2.0
Strength
Groups at similarity 0.8
FDR
3e-7
3e-6
3e-5
3e-4
3e-3
4e-2
Gene count
5
20
40
50

Figure S3. Gene Ontology enrichment analysis. Gene Ontology enrichment analysis of the 62 sensitization hits performed using the STRING platform. **a** Biological processes. **b** Molecular functions. **c** Cellular components.

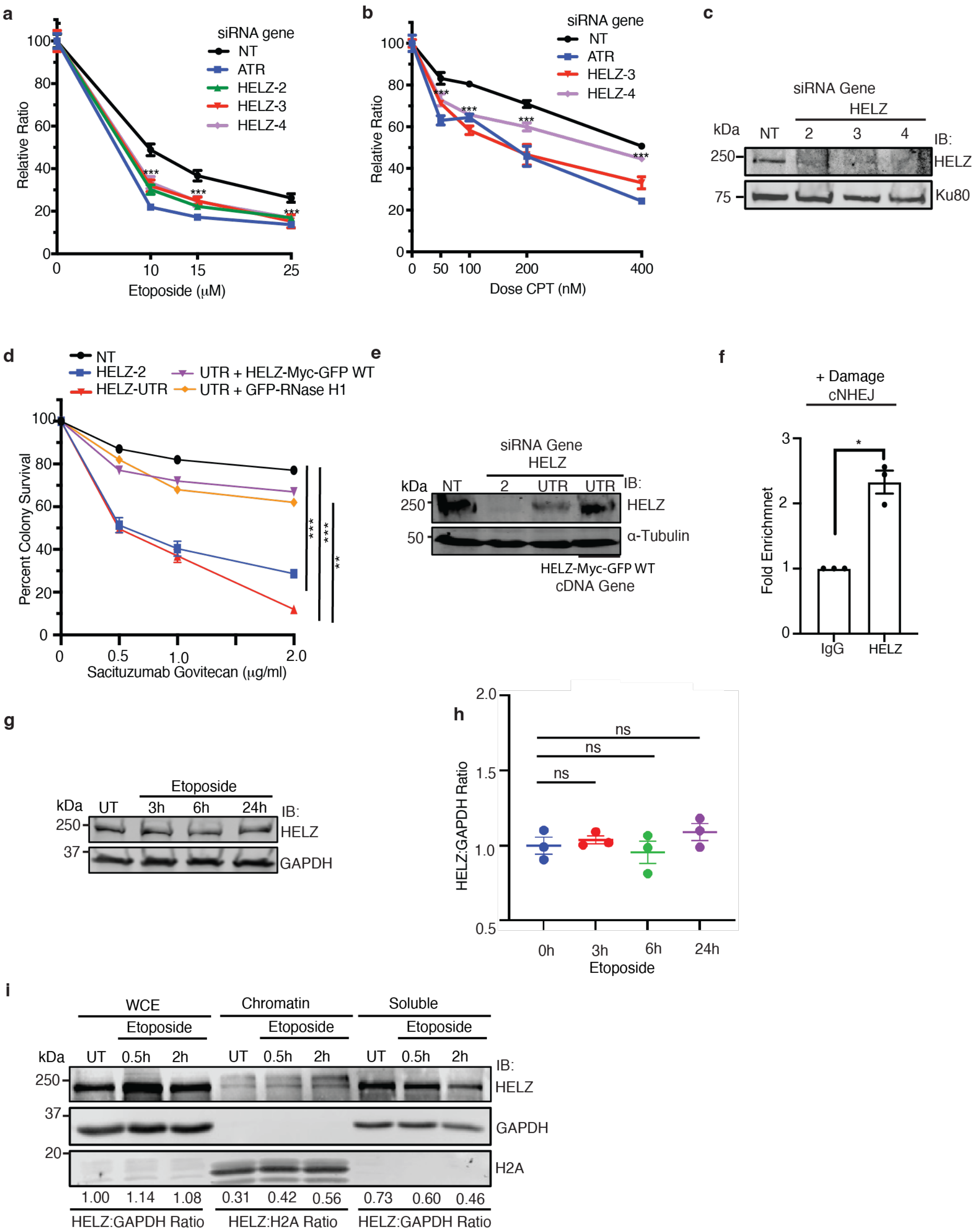
a
siRNA gene
NT
ATR
HELZ-2
HELZ-3
HELZ-4
Relative Ratio
Etoposide (μM)
b
siRNA gene
NT
ATR
HELZ-3
HELZ-4
Relative Ratio
Dose CPT (nM)
c
siRNA Gene
HELZ
kDa
NT
2
3
4
IB:
250
HELZ
75
Ku80
d
NT
HELZ-2
HELZ-UTR
UTR + HELZ-Myc-GFP WT
UTR + GFP-RNase H1
Percent Colony Survival
Sacituzumab Govitecan (μg/ml)
e
siRNA Gene
HELZ
kDa
NT
2
UTR
UTR
IB:
250
HELZ
50
α-Tubulin
HELZ-Myc-GFP WT
cDNA Gene
f
+ Damage
cNHEJ
Fold Enrichmnet
IgG
HELZ
g
Etoposide
kDa
UT
3h
6h
24h
IB:
250
HELZ
37
GAPDH
h
HELZ:GAPDH Ratio
ns
ns
ns
0h
3h
6h
24h
Etoposide
i
WCE
Chromatin
Soluble
Etoposide
Etoposide
Etoposide
kDa
UT
0.5h
2h
UT
0.5h
2h
UT
0.5h
2h
IB:
250
HELZ
37
GAPDH
20
H2A
1.00
1.14
1.08
0.31
0.42
0.56
0.73
0.60
0.46
HELZ:GAPDH Ratio
HELZ:H2A Ratio
HELZ:GAPDH Ratio

Figure S4. HELZ depletion causes hypersensitivity to DSB-inducing agents. a-b HELZ depletion causes hypersensitivity to etoposide (**a**) and CPT (**b**). H128 cells were transfected with siRNA targeting HELZ, ATR, or a NT control. 72 hours after transfection, cells were treated with etoposide or CPT for 72 hours prior to measuring metabolic activity. Mean ± SD, n = 3 independent experiments. **c** Western blot analysis of HELZ expression in H128 cells demonstrating HELZ knockdown. **d** BT-549 cells were transfected with indicated siRNA. 72 h after transfection, cells were treated with or without indicated doses of SG continuously. Mean ± SD, n = 3 independent experiments. **e** Western blot analysis showing HELZ knockdown and rescue in BT-549 cells from (**d**). **f** CHIP-qPCR analysis of endogenous HELZ enrichment at NHEJ prone DNA repair sites in DIvA cells. DSBs were induced by treatment with 500 nM 4-OHT for 4 h. Mean ± SD, n = 3 independent experiments. **g** Western blot analysis of HELZ expression level in U2OS cells after 20 μM etoposide treatment for indicated time points. **h** Quantification of HELZ:GAPDH ratio from (g). Mean ± SD, n = 3 independent experiments. **i** U2OS biochemical fractionation after 20 μM etoposide treatment (UT: untreated). Western blots are representative of three independent experiments. Statistical analyses used an unpaired two-tailed *t*-test (Fig f), or one-way ANOVA (Fig. a, b, and h) or two-way ANOVA (Fig. d) followed by Dunnett's multiple comparison test where appropriate. * $p < 0.05$, ** $p<0.01$, *** $p < 0.001$; ns, not significant. Source data are provided as a Source Data file

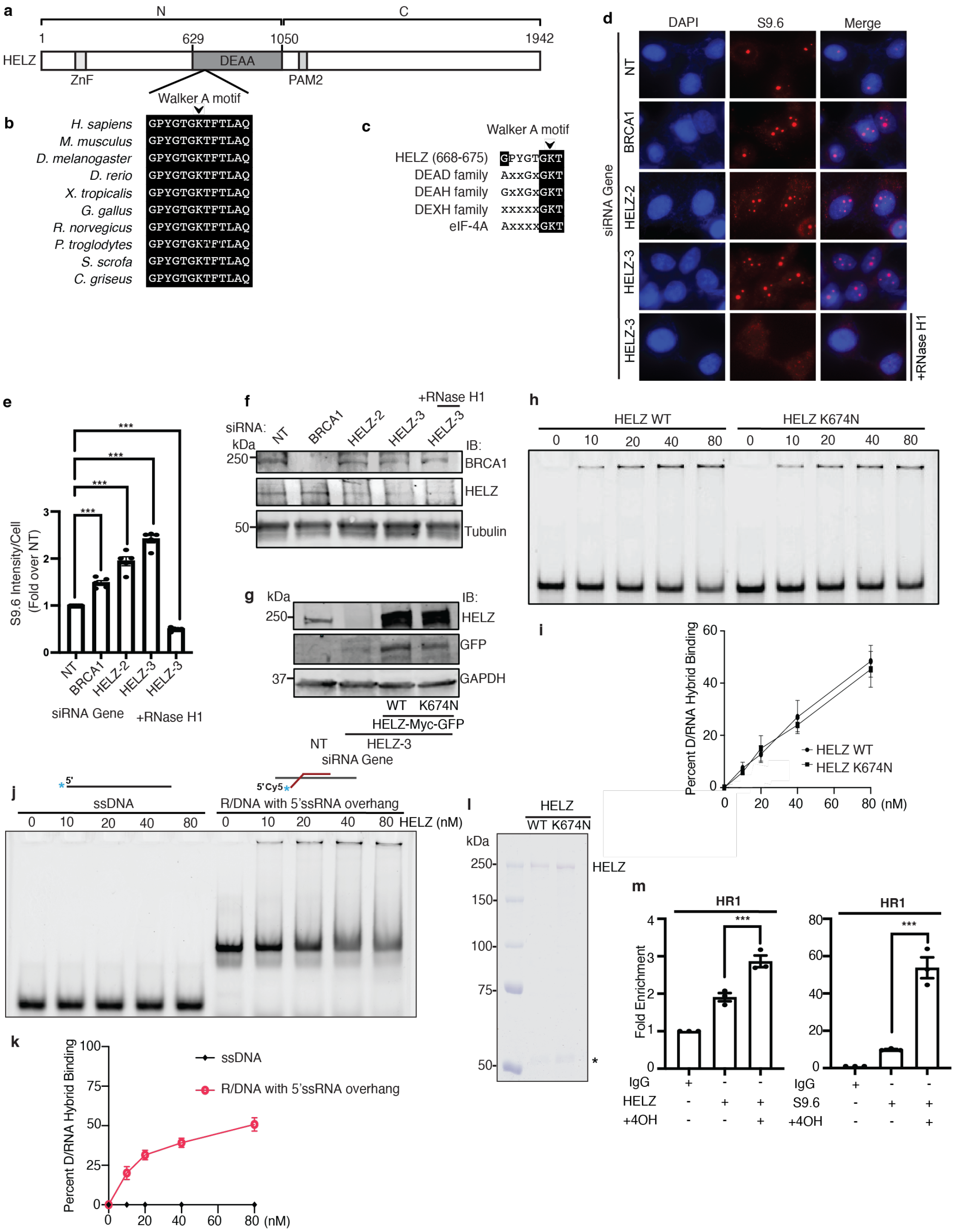
a
N
C
1
629
1050
1942
HELZ
DEAA
ZnF
PAM2
Walker A motif
b
H. sapiens
M. musculus
D. melanogaster
D. rerio
X. tropicalis
G. gallus
R. norvegicus
P. troglodytes
S. scrofa
C. griseus
GPYGTGKTFTLAQ
c
Walker A motif
HELZ (668-675) GPYGTGKT
DEAD family AxxGxGKT
DEAH family GxXGxGKT
DEXH family xxxxxGKT
eIF-4A AxxxxGKT
d
DAPI
S9.6
Merge
siRNA Gene
NT
BRCA1
HELZ-2
HELZ-3
HELZ-3
+RNase H1
e
S9.6 Intensity/Cell (Fold over NT)
***
NT
BRCA1
HELZ-2
HELZ-3
HELZ-3
siRNA Gene
+RNase H1
f
+RNase H1
siRNA:
kDa
NT
BRCA1
HELZ-2
HELZ-3
HELZ-3
250
50
IB:
BRCA1
HELZ
Tubulin
g
kDa
250
37
IB:
HELZ
GFP
GAPDH
WT
K674N
HELZ-Myc-GFP
NT
HELZ-3
siRNA Gene
h
HELZ WT
HELZ K674N
0 10 20 40 80
i
Percent D/RNA Hybrid Binding
HELZ WT
HELZ K674N
(nM)
j
5'
5'Cy5
ssDNA
R/DNA with 5'ssRNA overhang
0 10 20 40 80
HELZ (nM)
k
Percent D/RNA Hybrid Binding
ssDNA
R/DNA with 5'ssRNA overhang
(nM)
l
HELZ
WT K674N
kDa
250
150
100
75
50
HELZ
*
m
HR1
Fold Enrichment
***
IgG
HELZ
+4OH
S9.6

Figure S5. HELZ prevents R loop accumulation in cells. a Schematic representation of human HELZ indicating Zinc finger (ZnF), putative helicase (DEAA: Asp, Glu, Ala, Ala) domain, and polyA-binding protein (PABP) interacting motif 2 (PAM2). HELZ N- and C-terminal fragments are indicated above the scheme. **b** Sequence alignment of Walker A motif in different species. Strictly conserved residues are shown as white letters on black background. K674 is the residue mutated to N to abolish ATPase dependent helicase activity of HELZ. **c** Highly conserved amino acid regions in HELZ compared to DEAD, DEAH, DEXH families and eIF-4A protein. x indicates any amino acid. K in the Walker A motif is often the amino acid mutated to investigate the helicase activity of the protein in these families. **d-f** MDA-MB-231 cells were transfected with HELZ siRNA and processed 72 h later for indirect immunofluorescence with fixation, RNaseH treatment for 30 min, and anti-S9.6 antibody. Representative images (**d**) and quantification (**e**) are shown. The median is indicated by a horizontal line. (n = 5 independent experiments). **f** Western blot showing HELZ knockdown in MDA-MB-231 cells. **g** Western blot analysis showing HELZ knockdown and overexpression in HCT116 cells. **h-i** EMSA showing comparable binding of insect purified recombinant HELZ WT and K674N with RNA/DNA hybrid with 5' ssRNA overhang. Mean ± SD, n = 3 independent experiments. **j-k** EMSA showing binding of insect purified recombinant HELZ WT with RNA/DNA hybrid with 5' ssRNA overhang but not ssDNA. Mean ± SD, n = 3 independent experiments. **l** Coomassie blue gel of insect purified recombinant HELZ WT and K674N. **m** ChIP-qPCR analysis of endogenous HELZ and S9.6 enrichment at HR1 prone DNA repair sites. DSBs were induced by treating DIvA cells with 500 nM of 4-OHT for 4 h. Mean ± SD, n = 3 independent experiments. All western blots are representative of three independent experiments. Statistical analyses used two-way ANOVA (Fig. e and m) followed by Dunnett's

multiple comparison test. * $p < 0.05$, ** $p<0.01$, *** $p < 0.001$; ns, not significant. Source data are provided as a Source Data file

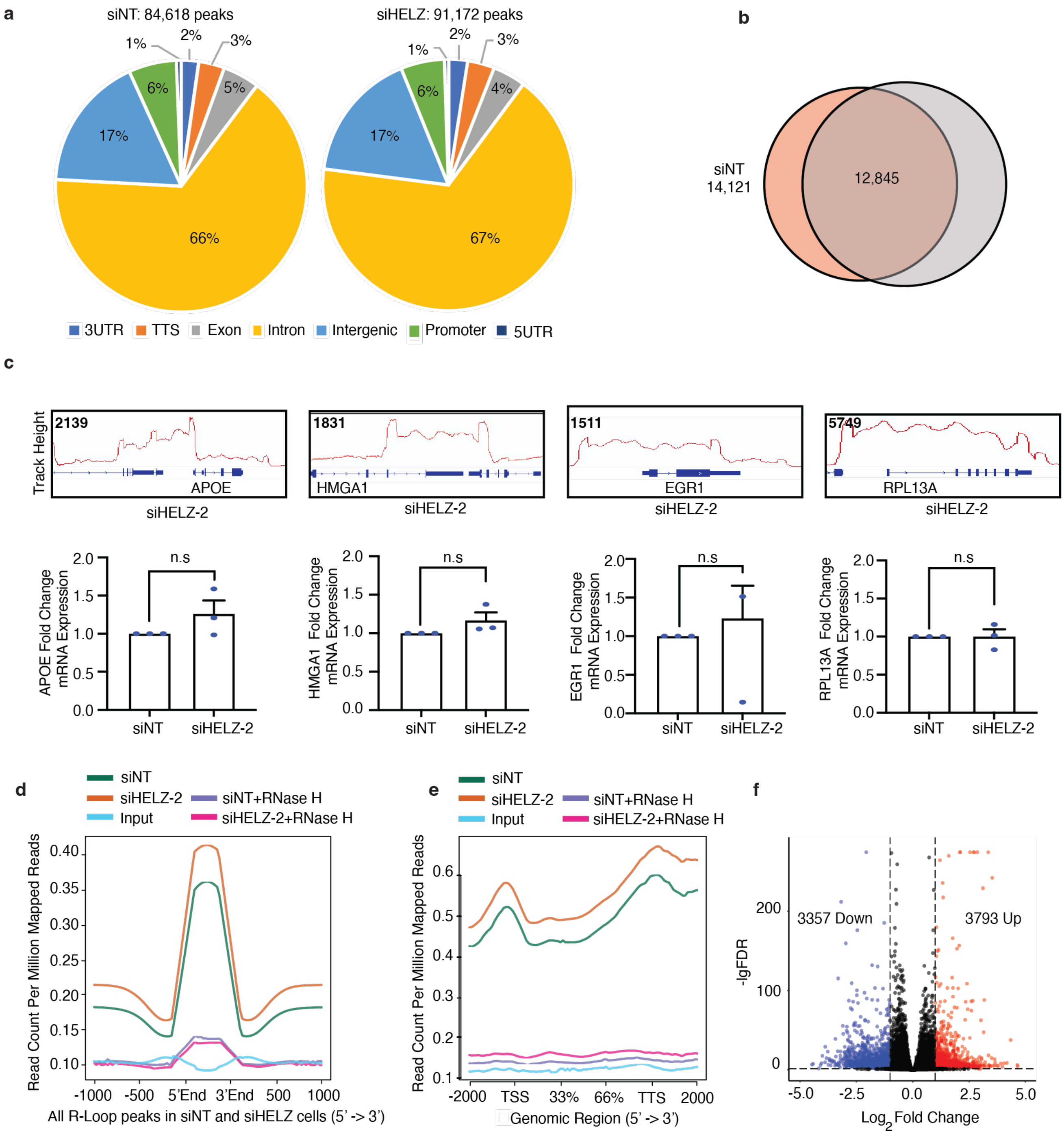

a
siNT: 84,618 peaks
siHELZ: 91,172 peaks
1%
2%
3%
5%
4%
6%
17%
66%
67%
3UTR
TTS
Exon
Intron
Intergenic
Promoter
5UTR
b
siNT
14,121
12,845
c
Track Height
2139
APOE
1831
HMGA1
1511
EGR1
5749
RPL13A
siHELZ-2
APOE Fold Change mRNA Expression
HMGA1 Fold Change mRNA Expression
EGR1 Fold Change mRNA Expression
RPL13A Fold Change mRNA Expression
n.s
siNT
siHELZ-2
d
siNT
siHELZ-2
Input
siNT+RNase H
siHELZ-2+RNase H
Read Count Per Million Mapped Reads
-1000
-500
5'End
3'End
500
1000
All R-Loop peaks in siNT and siHELZ cells (5' -> 3')
e
-2000
TSS
33%
66%
TTS
2000
Genomic Region (5' -> 3')
f
3357 Down
3793 Up
-lgFDR
Log2 Fold Change

Figure S6. HELZ depletion causes genome wide accumulation of R loops. a Genomic distribution of R loop regions identified in siNT and siHELZ cells. **b** Venn diagram of the number of genes annotated by R loop regions in siNT and siHELZ cells. **c** R loop upregulation at selected genes with associated mRNA detected by ChIP-qPCR **d** Ngsplot of R loop read counts across all R loop peaks identified in siNT and siHELZ cells. **e** Ngsplot of R loop read counts across defined human RefSeq (hg38) genes. **f** Volcano plot showing the number of R loop regions increased or decreased in HELZ depleted U2OS cells. The R loop regions that achieved an FDR of <0.05 and fold-change of ≥ 2 are indicated in red (increased) and blue (decreased). Statistical analyses used an unpaired two-tailed *t*-test (Fig. c). * $p < 0.05$, ** $p<0.01$, *** $p < 0.001$; ns, not significant.

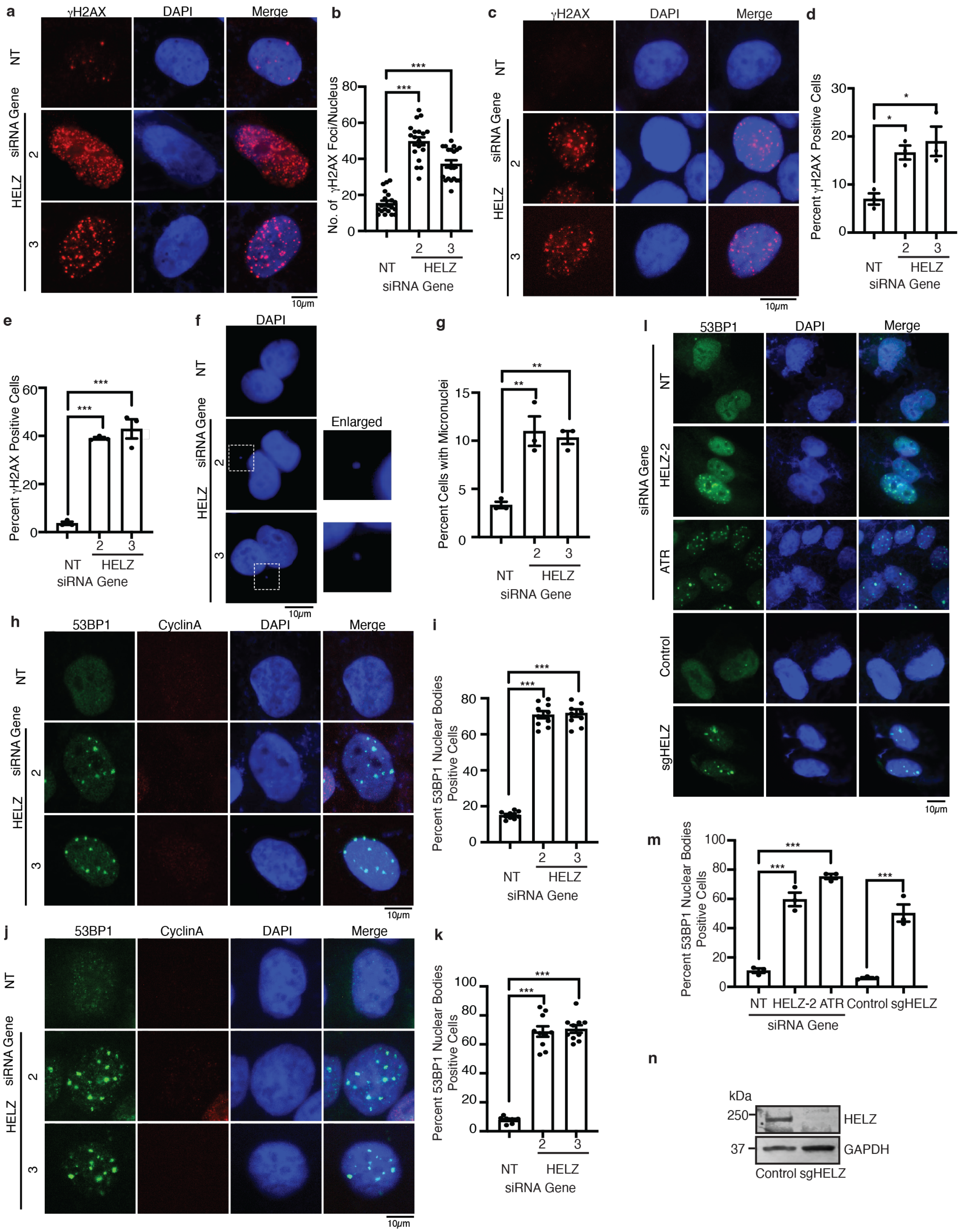
a
γH2AX
DAPI
Merge
NT
siRNA Gene
HELZ
2
3
10μm
b
No. of γH2AX Foci/Nucleus
80
60
40
20
0
***
***
NT
2
3
HELZ
siRNA Gene
c
γH2AX
DAPI
Merge
NT
siRNA Gene
HELZ
2
3
10μm
d
Percent γH2AX Positive Cells
30
20
10
0
*
*
NT
2
3
HELZ
siRNA Gene
e
Percent γH2AX Positive Cells
60
40
20
0
***
***
NT
2
3
HELZ
siRNA Gene
f
DAPI
Enlarged
NT
siRNA Gene
HELZ
2
3
10μm
g
Percent Cells with Micronuclei
15
10
5
0
**
**
NT
2
3
HELZ
siRNA Gene
l
53BP1
DAPI
Merge
NT
HELZ-2
ATR
siRNA Gene
Control
sgHELZ
10μm
h
53BP1
CyclinA
DAPI
Merge
NT
siRNA Gene
HELZ
2
3
10μm
i
Percent 53BP1 Nuclear Bodies Positive Cells
80
60
40
20
0
***
***
NT
2
3
HELZ
siRNA Gene
m
Percent 53BP1 Nuclear Bodies Positive Cells
100
80
60
40
20
0
***
***
***
NT
HELZ-2
ATR
Control
sgHELZ
siRNA Gene
j
53BP1
CyclinA
DAPI
Merge
NT
siRNA Gene
HELZ
2
3
10μm
k
Percent 53BP1 Nuclear Bodies Positive Cells
100
80
60
40
20
0
***
***
NT
2
3
HELZ
siRNA Gene
n
kDa
250
37
HELZ
GAPDH
Control
sgHELZ

Figure S7. HELZ deficiency causes genomic instability. a-d Spontaneous formation of γH2AX foci after 72 h of HELZ depletion in U2OS (**a-b**), RPE-1 cells (**c-d**), and HeLa (**e**). Representative images and quantification are shown. **f-g** Micronuclei formation in U2OS cells 72 h following HELZ depletion. Cells were fixed and stained with DAPI (**f**) and quantified (**g**). **h-k** Representative images and quantification from independent replicas for 53BP1 nuclear bodies in Cyclin A negative U2OS (**h-i**) and HeLa cells (**j-k**) silenced for HELZ or a NT control. **l-n** Immunofluorescence staining for spontaneous 53BP1 nuclear bodies was performed in U2OS cells depleted for HELZ or a NT control or in U2OS HELZ KO cells generated by CRISPR/Cas9. Representative images and quantification are shown. **m** Western blot showing KO of HELZ in U2OS cells. For Fig. **b**, **d**, **e**, **g**, **i**, **k**, **m**, mean ± SD, n = 3 independent experiments. Statistical analyses used one-way ANOVA followed by Dunnett's multiple comparison test. * $p < 0.05$, ** $p<0.01$, *** $p < 0.001$; ns, not significant. Source data are provided as a Source Data file

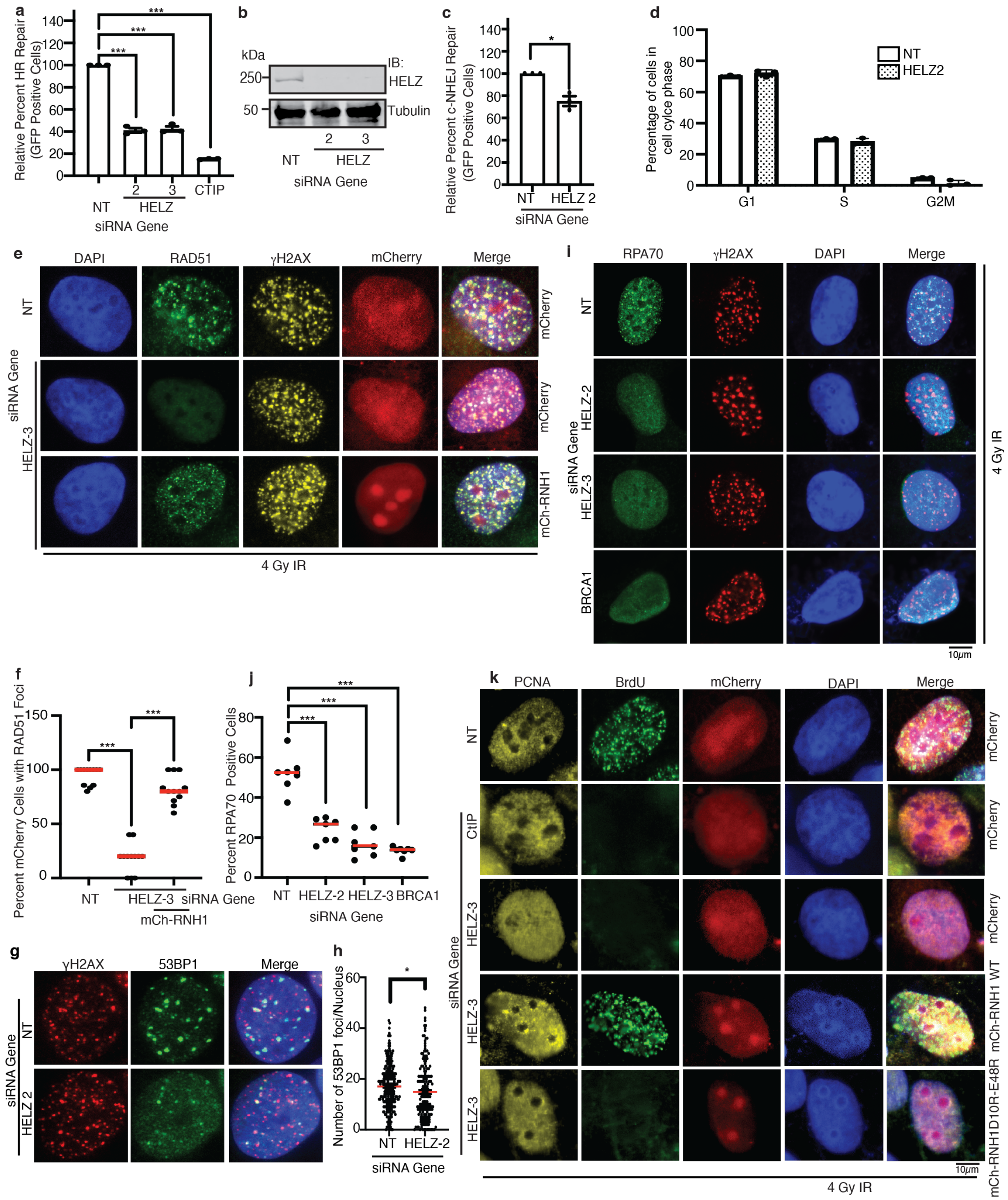

a
Relative Percent HR Repair (GFP Positive Cells)
NT
HELZ
2
3
CTIP
siRNA Gene
b
kDa
250
50
IB:
HELZ
Tubulin
siRNA Gene
c
Relative Percent c-NHEJ Repair (GFP Positive Cells)
HELZ 2
d
Percentage of cells in cell cylce phase
NT
HELZ2
G1
S
G2M
e
DAPI
RAD51
γH2AX
mCherry
Merge
HELZ-3
mCh-RNH1
4 Gy IR
i
RPA70
HELZ-2
BRCA1
10μm
f
Percent mCherry Cells with RAD51 Foci
j
Percent RPA70 Positive Cells
k
PCNA
BrdU
CtIP
mCh-RNH1 WT
mCh-RNH1 D10R-E48R
g
53BP1
HELZ 2
h
Number of 53BP1 foci/Nucleus

Figure S8. HELZ depletion impairs homologous recombination. a U2OS cells containing an integrated DR-GFP HR reporter were silenced with indicated siRNA and transfected with I-SceI endonuclease. Live cells collected were subjected to flow cytometry. GFP positive cells were gated to assess for HR. Mean ± SD, n = 3 independent experiments. **b** Western blot showing HELZ knockdown from **a**. **c** Classical non-homologous end joining (c-NHEJ) repair efficiency in HEK293 cells integrated with the EJ7-GFP reporter, transfected with indicated siRNAs and Cas9/sgRNA targeting the reporter locus Mean ± SD, n = 3 independent experiments. **d** Cell cycle analysis of U2OS cells following depletion of HELZ for 72 h and stained with propidium iodide Mean ± SD, n = 3 independent experiments. **e-f** U2OS cells were transfected with HELZ or NT siRNA control for 72h and mCherry-RNaseH1 or mCherry for 48h, treated with 4 Gy IR, and processed after 4h for indirect immunofluorescence with indicated antibodies. Representative images (**e**) and quantification are shown (n = 12 independent experiments) (**f**). The median is indicated by a horizontal line. **g-h** U2OS cells were silenced with HELZ or a NT control for 72h, treated with 4 Gy IR, and processed after 4h for indirect immunofluorescence with indicated antibodies. Representative images (**g**) and quantification from three independent experiments are shown (**h**). The median is indicated by a horizontal line. **i-j** U2OS cells were depleted for HELZ, BRCA1, or NT siRNA for 72h, treated with 4 Gy IR, and processed 4h later for indirect immunofluorescence with indicated antibodies. Representative images and quantitation are shown (n = 7 independent experiments). The median is indicated by a horizontal line. **k**. U2OS cells were transfected with HELZ, CtIP or NT siRNA control for 72h and mCherry-RNaseH1 WT or D10R/E48R for 48 h, treated with 4 Gy IR, and processed 4h later for indirect immunofluorescence with indicated antibodies (n = 3 independent experiments). Statistical analyses used an unpaired two-tailed *t*-test (fig c and h), or one-way ANOVA (Fig. a and j) or two-way ANOVA (Fig. f)

followed by Dunnett's multiple comparison test where appropriate. * $p < 0.05$, ** $p<0.01$, *** $p < 0.001$; ns, not significant. Source data are provided as a Source Data file

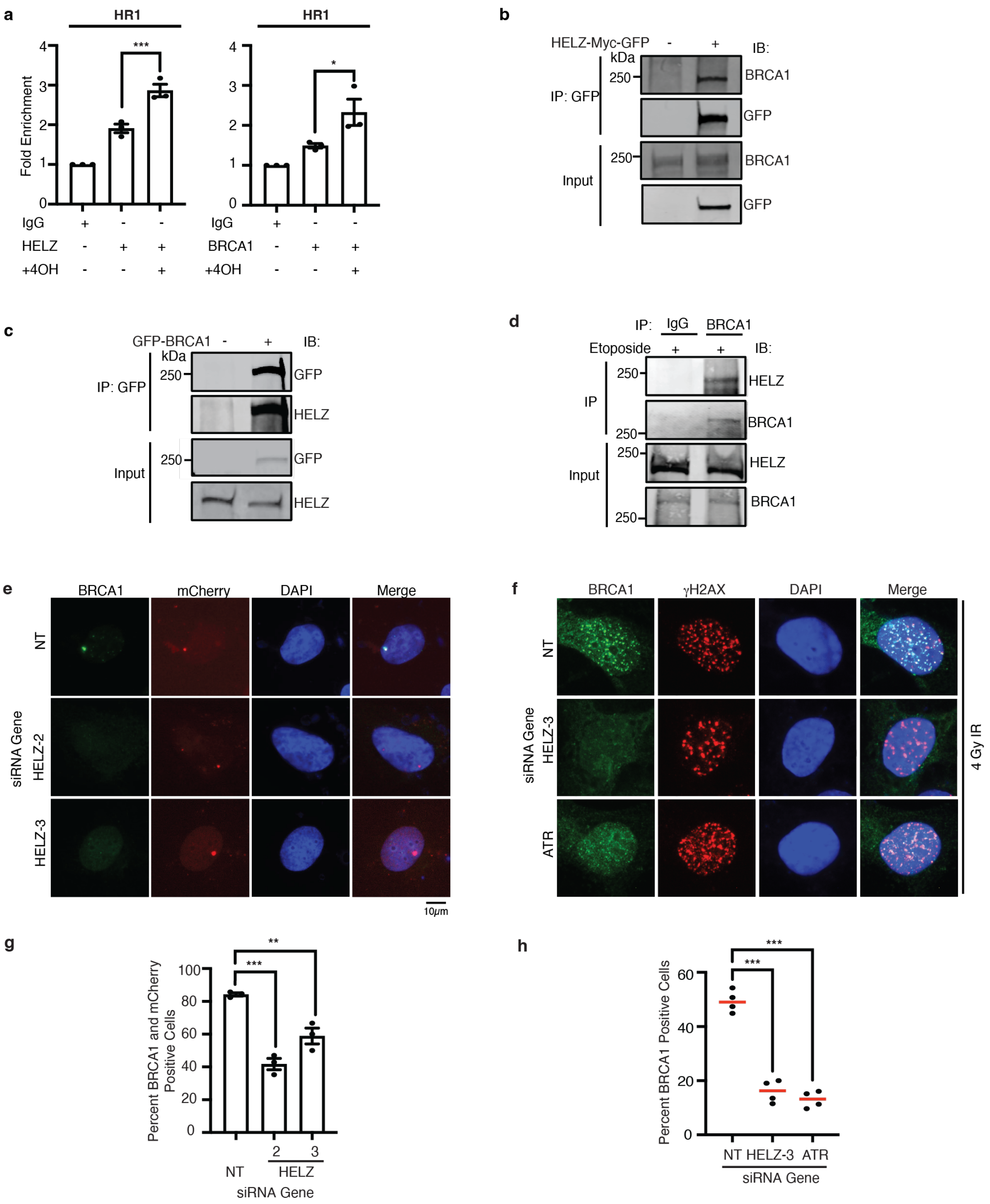
a
HR1
HR1
Fold Enrichment
IgG
HELZ
+4OH
BRCA1
b
HELZ-Myc-GFP
IB:
kDa
IP: GFP
Input
BRCA1
GFP
c
GFP-BRCA1
HELZ
d
IP:
IgG
Etoposide
IP
e
mCherry
DAPI
Merge
NT
siRNA Gene
HELZ-2
HELZ-3
10μm
f
γH2AX
ATR
4 Gy IR
g
Percent BRCA1 and mCherry Positive Cells
h
Percent BRCA1 Positive Cells

Figure S9. HELZ interacts with BRCA1 in a damage regulated and RNA-dependent manner and facilitates BRCA1 recruitment to DSBs by preventing accumulation of R loops. a ChIP-qPCR analysis of endogenous HELZ and BRCA1 enrichment at HR1 prone DNA repair sites in DIvA cells. DSBs were induced by treatment with 500 nM of 4-OHT for 4 h. Mean ± SEM, n = 3 independent experiments. **b-c** Co-IP of HELZ-Myc-GFP or GFP-BRCA1 expressed in HEK293T cells pulls down endogenous BRCA1 or HELZ respectively. **d** Co-IP of endogenous BRCA1 expressed in HEK293T cells treated with 20 μM etoposide for 4 h pulls down endogenous HELZ. **e-f** U2OS-265 Fok1 cells were depleted for HELZ. After 72 h, DSBs were induced by Shield-1 and 4-OHT. Cells were fixed after 4 h and stained with indicated antibodies. Percentage of cells with both a single red focus and green focus of BRCA1 were quantified. Representative images and quantification are shown. Mean ± SD, n = 3 independent experiments shown. **g-h** U2OS cells were transfected with HELZ, ATR, or NT siRNA, treated with 4 Gy IR, and fixed after 4 h for indirect immunofluorescence with indicated antibodies. Representative images and quantification from three independent experiments are shown. The median is indicated. Western blots are representative of three independent experiments. Statistical analyses used one-way ANOVA (Fig g and h) or two-way ANOVA (Fig. a) followed by Dunnett's multiple comparison test * $p < 0.05$, ** $p<0.01$, *** $p < 0.001$; ns, not significant. Source data are provided as a Source Data file

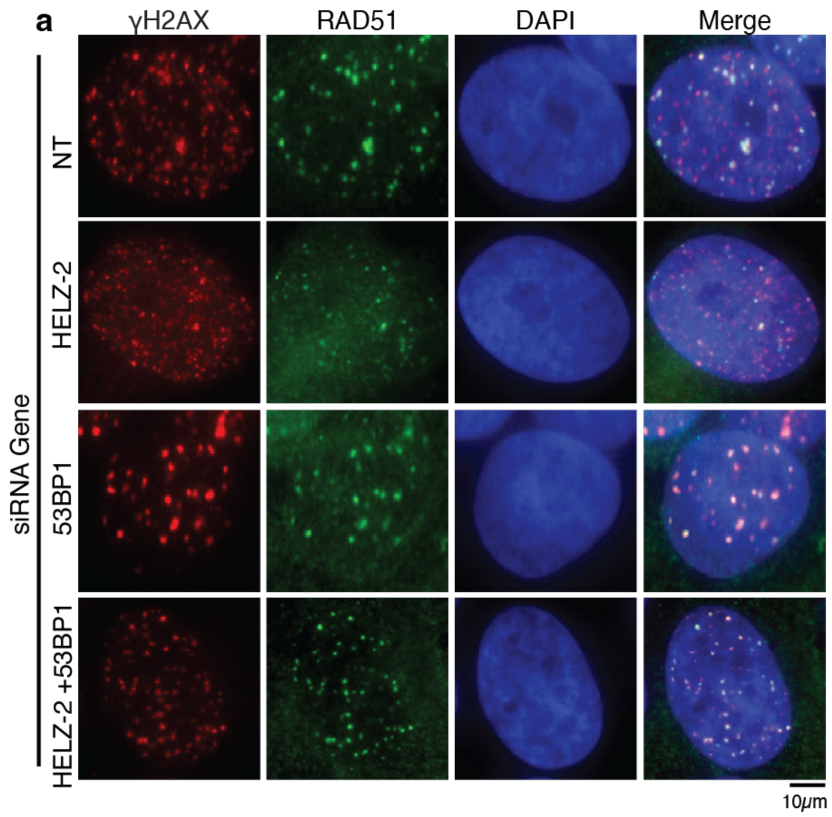
a
γH2AX
RAD51
DAPI
Merge
siRNA Gene
NT
HELZ-2
53BP1
HELZ-2 +53BP1
10μm

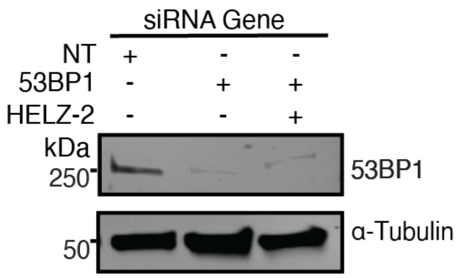
b
siRNA Gene
NT + - -
53BP1 - + +
HELZ-2 - - +
kDa
250
50
53BP1
α-Tubulin

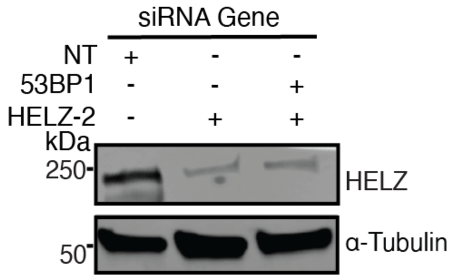
c
siRNA Gene
NT + - -
53BP1 - - +
HELZ-2 - + +
kDa
250
50
HELZ
α-Tubulin

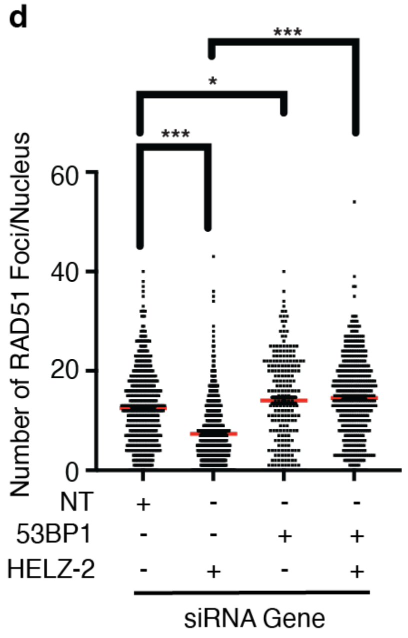
d
***
*
***
Number of RAD51 Foci/Nucleus
60
40
20
0
NT + - - -
53BP1 - - + +
HELZ-2 - + - +
siRNA Gene

Figure S10. 53BP1 depletion rescues the impairment in IR-induced RAD51 foci of HELZ depletion. a-d U2OS cells were silenced with HELZ, 53BP1, or a NT control for 72h, treated with 4 Gy IR, and processed after 4h for indirect immunofluorescence with indicated antibodies. Representative images (**a**) and quantification from three independent replicas are shown (**d**). The median is indicated by a horizontal line**. b-c** Western blot showing HELZ and 53BP1 knockdown in U2OS cells in (**a**). Western blots are representative of three independent experiments. Statistical analyses used two-way ANOVA followed by Dunnett's (Fig. d) multiple comparison test. * $p < 0.05$, ** $p<0.01$, *** $p < 0.001$; ns, not significant. Source data are provided as a Source Data file

Supplementary Data 1

Provided as excel file

Supplementary Data 2

Provided as excel file
