# Supplementary material for: HELZ is a RNA-DNA helicase that resolves R loops to facilitate homologous recombination repair
Source: Nat Commun. 2026 Jul 23;17:6968. doi: 10.1038/s41467-026-75089-3 (PMC13396810; doi:10.1038/s41467-026-75089-3)
Supplement: Supplementary file 2 — Description of Additional Supplementary Files [file 41467_2026_75089_MOESM2_ESM.pdf]

Description of Additional Supplementary Files

File Name: Supplementary Data 1

Description: Primary Etoposide Hypersensitivity siRNA Screen

File Name: Supplementary Data 2

Description: Positive Hits from Primary Etoposide Hypersensitivity siRNA Screen (62 hits)
