# Supplementary material for: HELZ is a RNA-DNA helicase that resolves R loops to facilitate homologous recombination repair
Source: Nat Commun. 2026 Jul 23;17:6968. doi: 10.1038/s41467-026-75089-3 (PMC13396810; doi:10.1038/s41467-026-75089-3)
Supplement: Supplementary file 5 — Reporting Summary [file 41467_2026_75089_MOESM5_ESM.pdf]

nature portfolio

Corresponding author(s): Yu, David

Last updated by author(s): Jul 24, 2025

Reporting Summary

Nature Portfolio wishes to improve the reproducibility of the work that we publish. This form provides structure for consistency and transparency in reporting. For further information on Nature Portfolio policies, see our Editorial Policies and the Editorial Policy Checklist.

Statistics

For all statistical analyses, confirm that the following items are present in the figure legend, table legend, main text, or Methods section.

n/a	Confirmed	
☐	☒	The exact sample size (*n*) for each experimental group/condition, given as a discrete number and unit of measurement
☐	☒	A statement on whether measurements were taken from distinct samples or whether the same sample was measured repeatedly
☐	☒	The statistical test(s) used AND whether they are one- or two-sided<br>*Only common tests should be described solely by name; describe more complex techniques in the Methods section.*
☐	☒	A description of all covariates tested
☐	☒	A description of any assumptions or corrections, such as tests of normality and adjustment for multiple comparisons
☐	☒	A full description of the statistical parameters including central tendency (e.g. means) or other basic estimates (e.g. regression coefficient) AND variation (e.g. standard deviation) or associated estimates of uncertainty (e.g. confidence intervals)
☐	☒	For null hypothesis testing, the test statistic (e.g. *F*, *t*, *r*) with confidence intervals, effect sizes, degrees of freedom and *P* value noted<br>*Give P values as exact values whenever suitable.*
☒	☐	For Bayesian analysis, information on the choice of priors and Markov chain Monte Carlo settings
☒	☐	For hierarchical and complex designs, identification of the appropriate level for tests and full reporting of outcomes
☒	☐	Estimates of effect sizes (e.g. Cohen's *d*, Pearson's *r*), indicating how they were calculated

Our web collection on statistics for biologists contains articles on many of the points above.

Software and code

Policy information about availability of computer code

Data collection	Qubit, FlowJo<br>Western -LiCor Odyssey System
Data analysis	SAMtools, Bowtie2 version 2.4.4, MACS2 version 2.2.6, DESeq2, annotatePeaks.pl from HOMER version 4.11

For manuscripts utilizing custom algorithms or software that are central to the research but not yet described in published literature, software must be made available to editors and reviewers. We strongly encourage code deposition in a community repository (e.g. GitHub). See the Nature Portfolio guidelines for submitting code & software for further information.

Data

Policy information about availability of data

All manuscripts must include a data availability statement. This statement should provide the following information, where applicable:

- Accession codes, unique identifiers, or web links for publicly available datasets
- A description of any restrictions on data availability
- For clinical datasets or third party data, please ensure that the statement adheres to our policy

All data are available in the main text or the supplementary materials. DRIPseq GEO accession number GSE242527

Research involving human participants, their data, or biological material

Policy information about studies with human participants or human data. See also policy information about sex, gender (identity/presentation), and sexual orientation and race, ethnicity and racism.

Reporting on sex and gender	N/A
Reporting on race, ethnicity, or other socially relevant groupings	N/A
Population characteristics	N/A
Recruitment	N/A
Ethics oversight	N/A

Note that full information on the approval of the study protocol must also be provided in the manuscript.

Field-specific reporting

Please select the one below that is the best fit for your research. If you are not sure, read the appropriate sections before making your selection.

☒ Life sciences ☐ Behavioural & social sciences ☐ Ecological, evolutionary & environmental sciences

For a reference copy of the document with all sections, see nature.com/documents/nr-reporting-summary-flat.pdf

Life sciences study design

All studies must disclose on these points even when the disclosure is negative.

Sample size	Sample sizes were chosen based on prior experience with these assays, published literature, and practical considerations regarding experimental feasibility. Each experiment was independently repeated at least three times, and the observed effect sizes were reproducible across independent experiments. No statistical method was used to predetermine sample size.
Data exclusions	No data were excluded from the analyses
Replication	Experiments were reproduced with at least 3 replicas. Experiments were selectively reproduced by multiple lab personnel.
Randomization	Cell in culture were treated with specific conditions/treatments and thus was not random. Analysis was randomized and validated by multiple lab personnel. Since the study does not involve patient or animal work but involves specific treatments of cultured cells and their outcome, randomization is not fully relevant to the study.
Blinding	Cells in culture needed to be treated for specific conditions so this could not be blinded; however, analysis was blinded/random amnd validated by multiple lab personnel.

Reporting for specific materials, systems and methods

We require information from authors about some types of materials, experimental systems and methods used in many studies. Here, indicate whether each material, system or method listed is relevant to your study. If you are not sure if a list item applies to your research, read the appropriate section before selecting a response.

Materials & experimental systems

n/a	Involved in the study	
☐	☒	Antibodies
☐	☒	Eukaryotic cell lines
☒	☐	Palaeontology and archaeology
☒	☐	Animals and other organisms
☒	☐	Clinical data
☒	☐	Dual use research of concern
☒	☐	Plants

Methods

n/a	Involved in the study	
☒	☐	ChIP-seq
☐	☒	Flow cytometry
☒	☐	MRI-based neuroimaging

Antibodies

Antibodies used

Primary antibodies used for western blotting, immunoprecipitation (IP) and immunofluorescence (IF) are as follows: HELZ (ThermoFisher customized 1:50 for Western; Proteintech # 26635-1-AP; 1:1000 for Western, 1.2 mL per 1 mg DNA for CHIP, 1:1000 for PLA); S9.6 (Millipore #MABE1095; 1:600 for Slot Blot, 1:250 for IF, 0.5 mL per one million cell nuclei for DRIP-WB, 5U per 1 mg DNA for DRIP) ; ssDNA (Millipore #MAB3868; 1:1000 for Slot Blot); IgG (Invitrogen # 10500C and Sigma # N103 for ChIP); GFP (Santa Cruz Tech # SC996; 1:1000 for Western; and abcam # ab290; 1:5000 for Western, 1 mg per 2 mg lysate for IP); γH2AX (Cell Signaling Technology # 2577S; 1:500 for IF; Millipore # 05-636; 1:6000 for IF; and Active Motif # 39117; 1 mL per 1 mg DNA for CHIP); KU80 (abcam # 80592; 1:1000 for Western); GAPDH (Santa Cruz tech # sc-47724; 1:1000 for Western); 53BP1 (Bethyl # A300-273A; 1:1000 for IF); RPA32 (Santa Cruz Tech # sc-14692; 1:400 for Western); pRPA32 S4/8 (Bethyl # A700-009; 1:1000 for Western); RPA70 (Cell Signaling # 2267S; 1:120 for IF); BRCA1 (Millipore # 07-434; 1:1000 for Western); CtIP (Millipore # MABE1060; 1:1000 for Western); Senataxin (Novus biological # NB100-57542; 1:1000 for IF) Rad51 (abcam # ab176458; 1:1000 for IF); Lamin A/C (Cell Signaling #2032; 1:1000 for Western) RFP (abcam # ab125244; 1:1000 for Western); α-Tubulin (Sigma # T6074; 1:10,000 for Western); and BrdU (BD Biosciences # 347580; 1:200 for IF). Secondary antibodies used for Western (at 1:1000) are: donkey anti-rabbit IR Dye 800 (Licor Biosciences #926-32213); donkey anti-rabbit IR Dye 680 (Licor Biosciences # 926-68023); donkey anti-mouse IR Dye 800 (Licor Biosciences # 926-32213); donkey anti-mouse IR Dye 680 (Licor Biosciences # 926-68022). Secondary antibodies used for IF (at 1:1000) are: goat anti-mouse Alexa Fluor 555 (Invitrogen # A21424); goat anti-rabbit Alexa Fluor 488 (Invitrogen # A11034); goat anti-mouse Alexa Fluor 647 (Invitrogen # A21235).

Validation

Commercial antibodies were validated by the respective manufacturers. Custom antibody of HELZ was validated by siRNA knockdown to demonstrate specificity. Fig 1e, g

Eukaryotic cell lines

Policy information about cell lines and Sex and Gender in Research

Cell line source(s)

Human SCLC line NCI-H128 was provide by the laboratory of Dr. Taofeek Owonikoko and grown in RPMI 1640 (Gibco) with 7.5% FBS. HEK293T, HCT116, HeLa, U20S, and MDA-MB-231 mammalian cell lines were purchased from American Type Culture Collection (ATCC, Manassas, VA). U20S-235 mCherry-LacI-Fok1 cell line were provided by Dr. Roger Greenberg. and U20S-DR-GFP, HEK293-DR-GFP and HEK293-EJ7-GFP cell lines were obtained from Dr. Jeremy Stark. AsiSI-ER-U2OS (DIvA-DSB inducible via AsiSI) cells were provided by Dr. Gaëlle Legube.

Authentication

Cell lines have been validated using multiple methodologies, including morphology, growth curve analysis, and STR profiling.

Mycoplasma contamination

All cell lines tested negative for mycoplasma contamination.

Commonly misidentified lines
(See ICLAC register)

N/A

Plants

Seed stocks

N/A

Novel plant genotypes

N/A

Authentication

N/A

Flow Cytometry

Plots

Confirm that:

☒ The axis labels state the marker and fluorochrome used (e.g. CD4-FITC).

☒ The axis scales are clearly visible. Include numbers along axes only for bottom left plot of group (a 'group' is an analysis of identical markers).

☒ All plots are contour plots with outliers or pseudocolor plots.

☒ A numerical value for number of cells or percentage (with statistics) is provided.

Methodology

Sample preparation

Cells were knocked down for the gene of interest. 72 h later, they were
harvested, washed and resuspended in cold PBS followed by fixation with cold 70% ethanol on

ice for a minimum of 1 h. After washing twice in PBS, cells were treated with RNase A and stained with propidium iodide at room temperature for a minimum of 30 min. Finally, at least 25,000 cells were analyzed for propidium iodide fluorescence on a flow cytometer (Cytek Aurora). Debris and aggregates were excluded throughout analysis by FlowJo software.

Instrument	Cytek Aurora
Software	FlowJo
Cell population abundance	At least 25,000 cells were sorted per sample
Gating strategy	At least 25,000 cells were analyzed for propidium iodide fluorescence on a flow cytometer (Cytek Aurora). Debris and aggregates were excluded throughout analysis by FlowJo software.

☐ Tick this box to confirm that a figure exemplifying the gating strategy is provided in the Supplementary Information.
