# Supplementary material for: HELZ is a RNA-DNA helicase that resolves R loops to facilitate homologous recombination repair
Source: Nat Commun. 2026 Jul 23;17:6968. doi: 10.1038/s41467-026-75089-3 (PMC13396810; doi:10.1038/s41467-026-75089-3)
Supplement: Supplementary file 7 — Source Data [file 41467_2026_75089_MOESM7_ESM.zip › Source_data_uncropped_blots.pptx]

## Slide 1
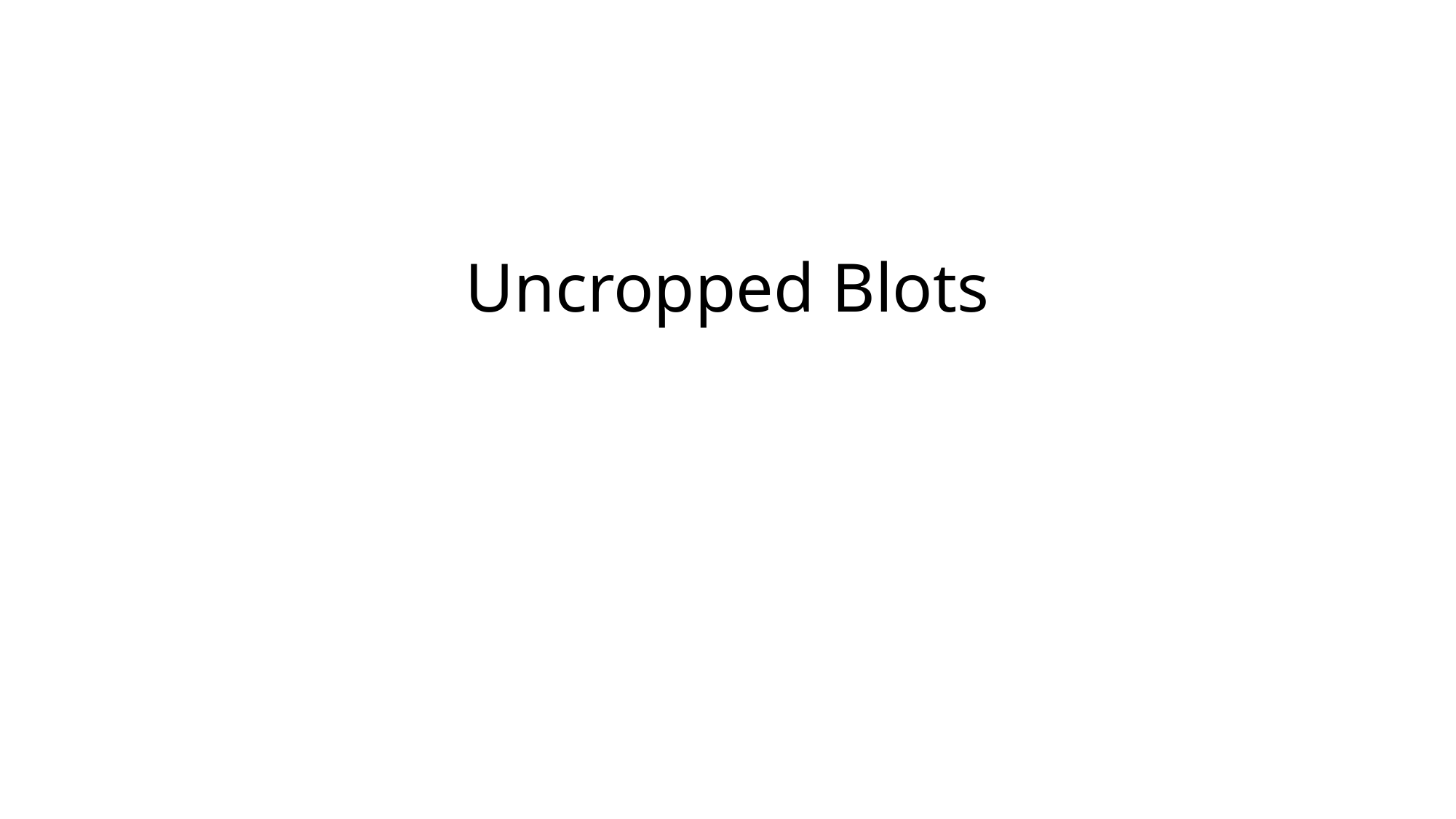

# Uncropped Blots

## Slide 2
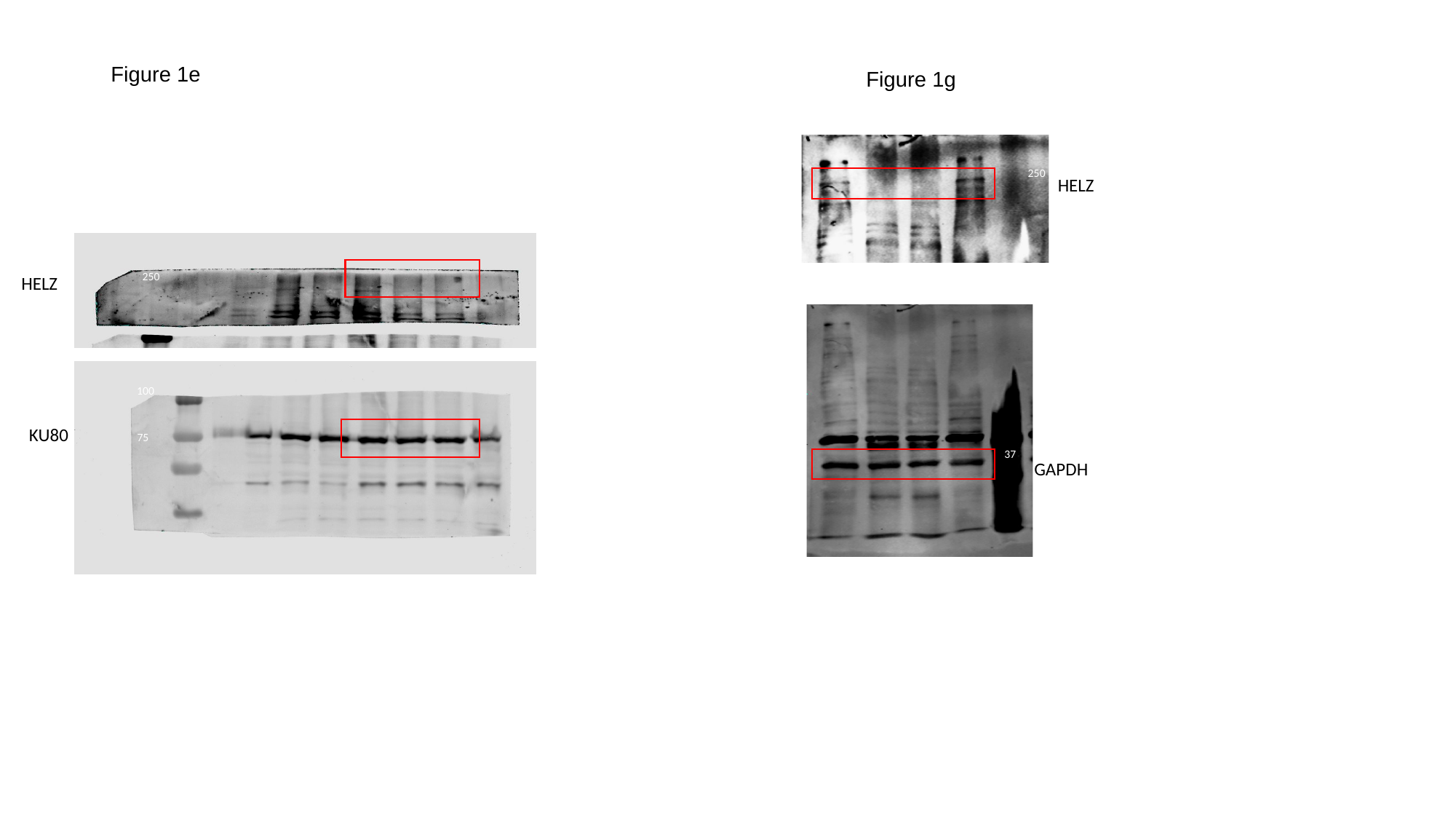

# Figure 1e
Figure 1g
250
HELZ
250
HELZ
100
KU80
75
37
GAPDH

## Slide 3
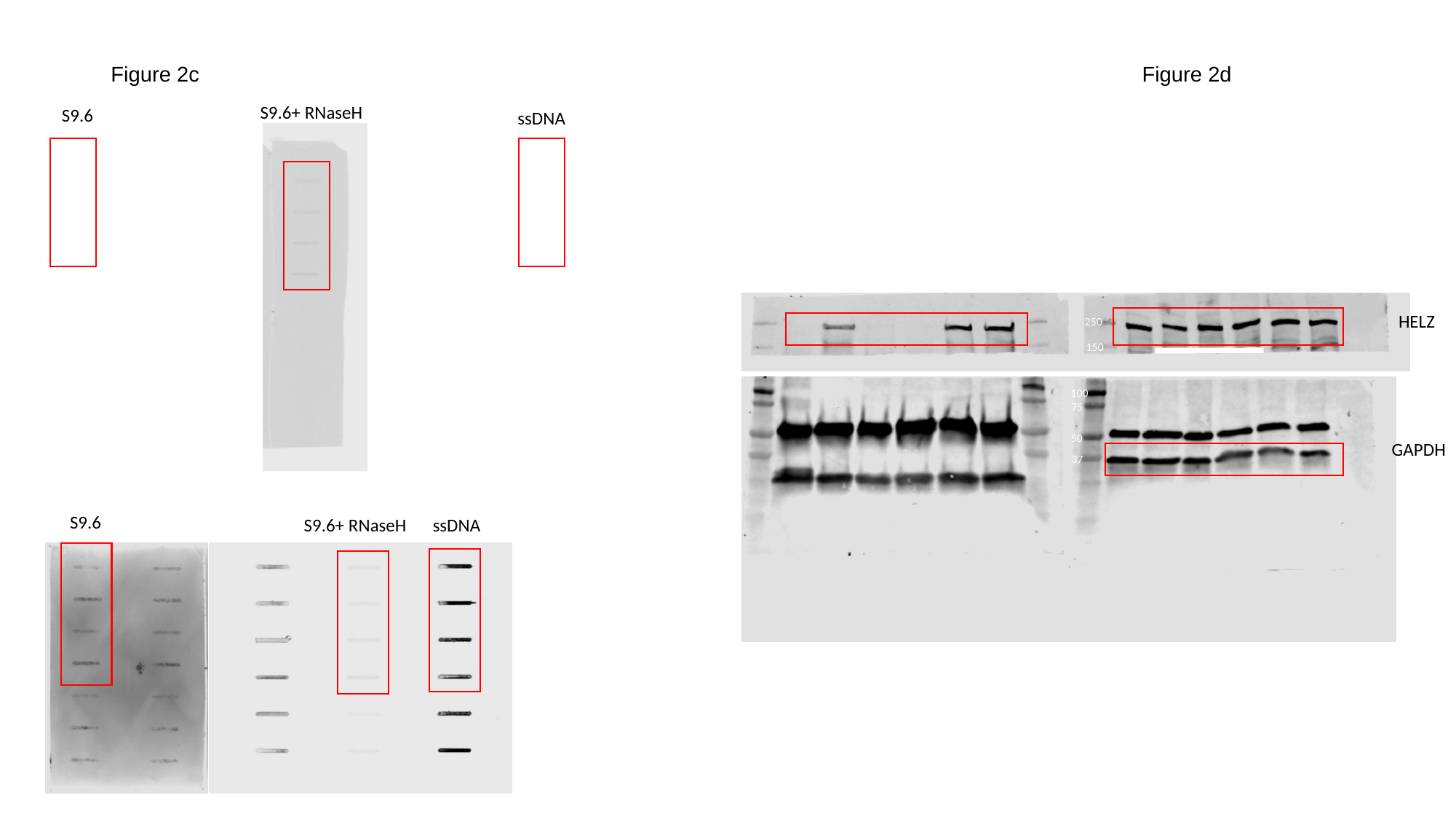

# Figure 2c
Figure 2d
S9.6+ RNaseH
S9.6
ssDNA
HELZ
250
150
100
75
50
GAPDH
37
S9.6
S9.6+ RNaseH
ssDNA

## Slide 4
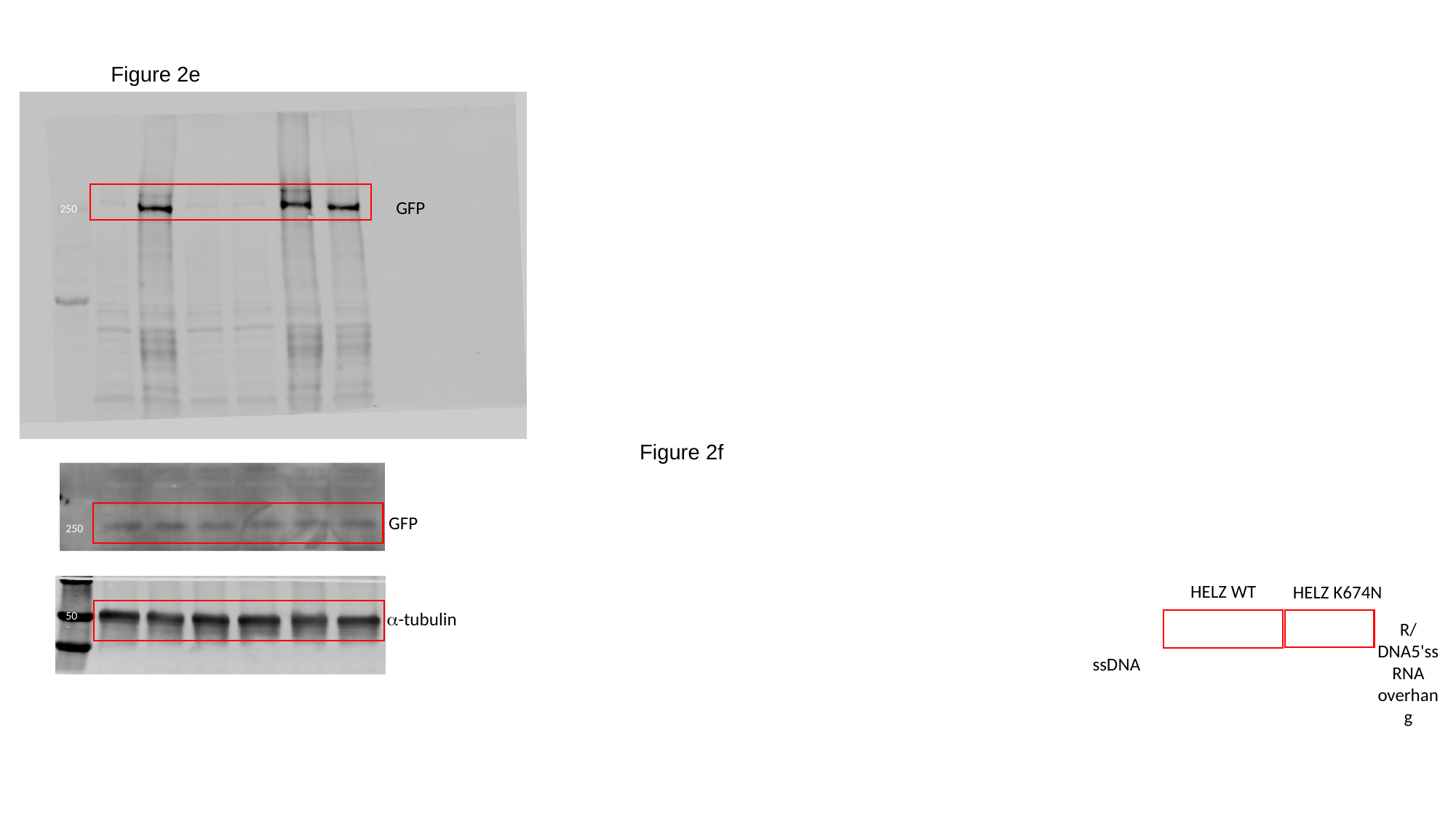

# Figure 2e
GFP
250
Figure 2f
GFP
250
HELZ WT
HELZ K674N
a-tubulin
50
R/DNA5'ssRNA overhang
ssDNA

## Slide 5
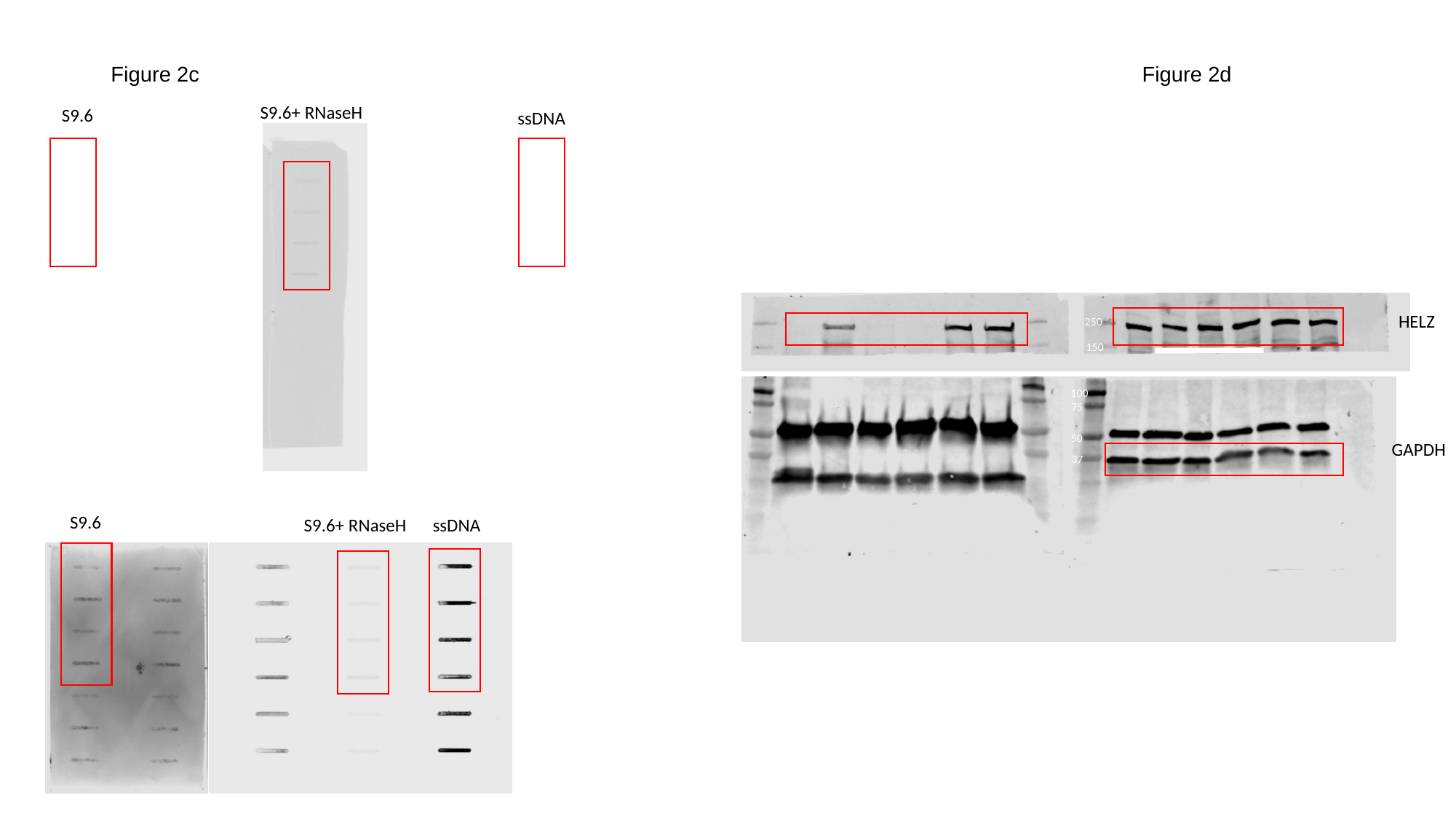

# Figure 2c
Figure 2d
S9.6+ RNaseH
S9.6
ssDNA
HELZ
250
150
100
75
50
GAPDH
37
S9.6
S9.6+ RNaseH
ssDNA

## Slide 6
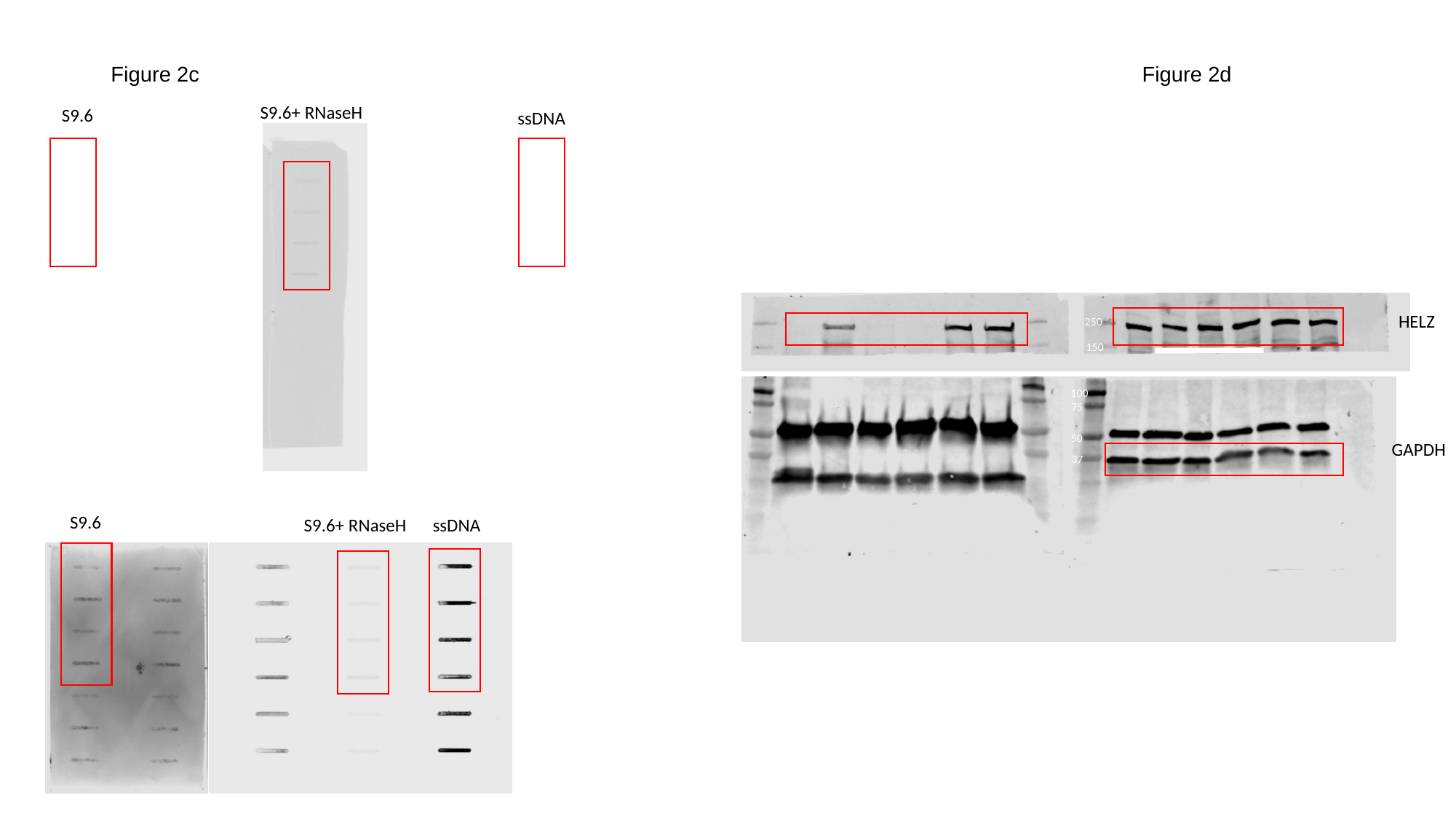

# Figure 2c
Figure 2d
S9.6+ RNaseH
S9.6
ssDNA
HELZ
250
150
100
75
50
GAPDH
37
S9.6
S9.6+ RNaseH
ssDNA

## Slide 7
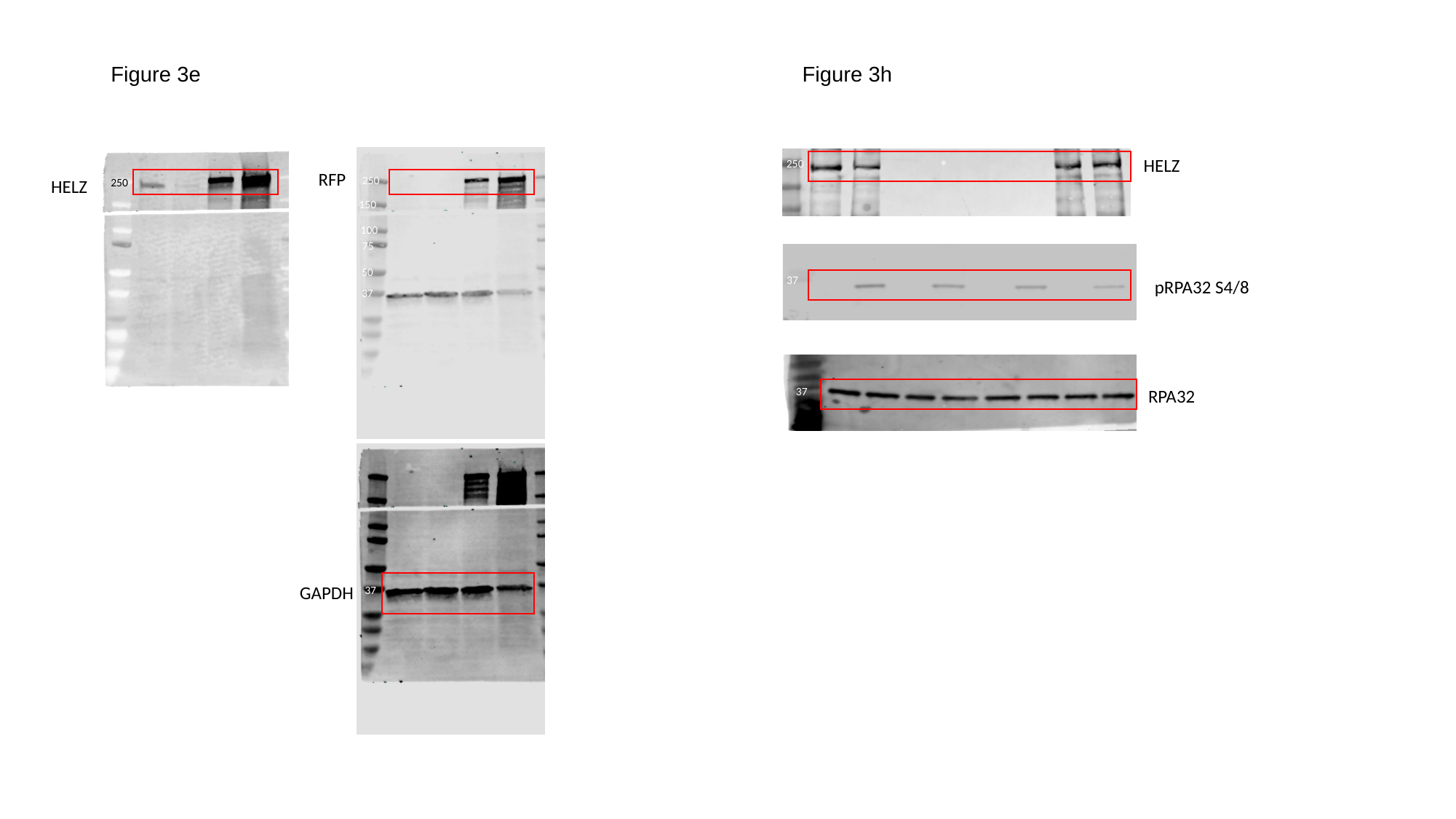

Figure 3e
Figure 3h
HELZ
250
RFP
250
250
HELZ
150
100
75
50
37
pRPA32 S4/8
37
37
RPA32
GAPDH
37

## Slide 8
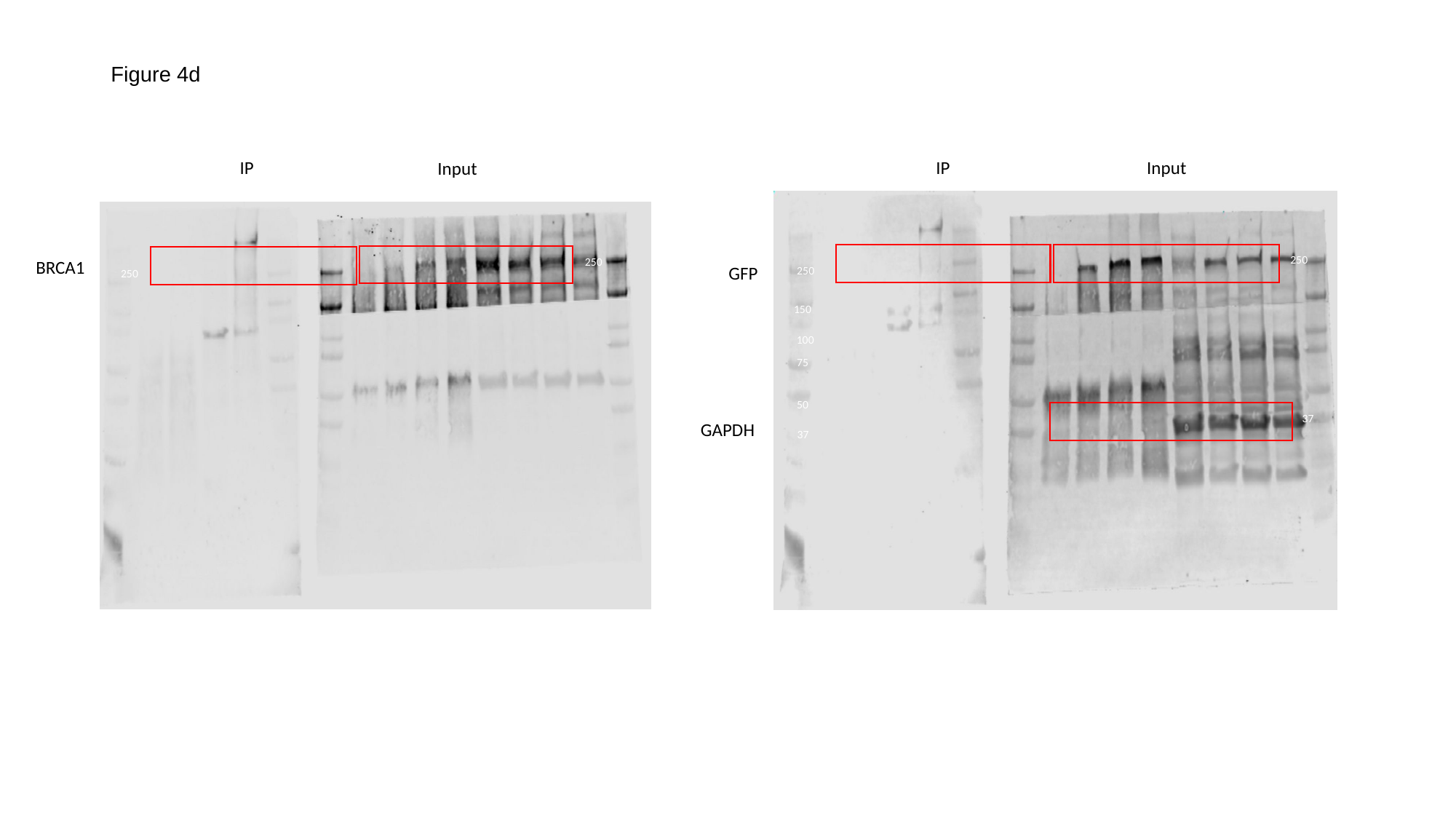

# Figure 4d
IP
IP
Input
Input
250
250
BRCA1
GFP
250
250
150
100
75
50
37
GAPDH
37

## Slide 9
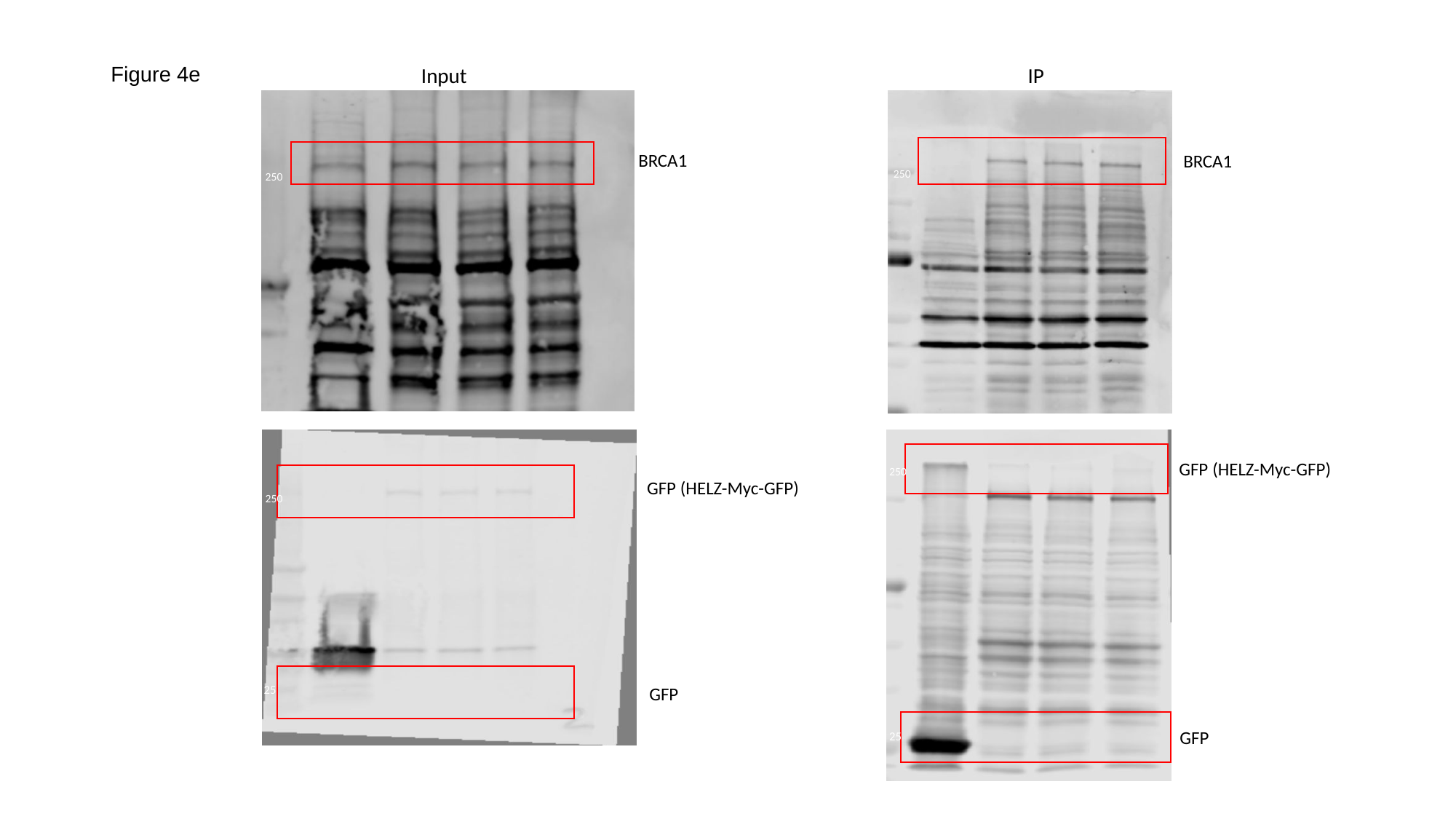

# Figure 4e
Input
IP
BRCA1
BRCA1
250
250
37
GFP (HELZ-Myc-GFP)
250
GFP (HELZ-Myc-GFP)
250
GFP
25
GFP
25

## Slide 10
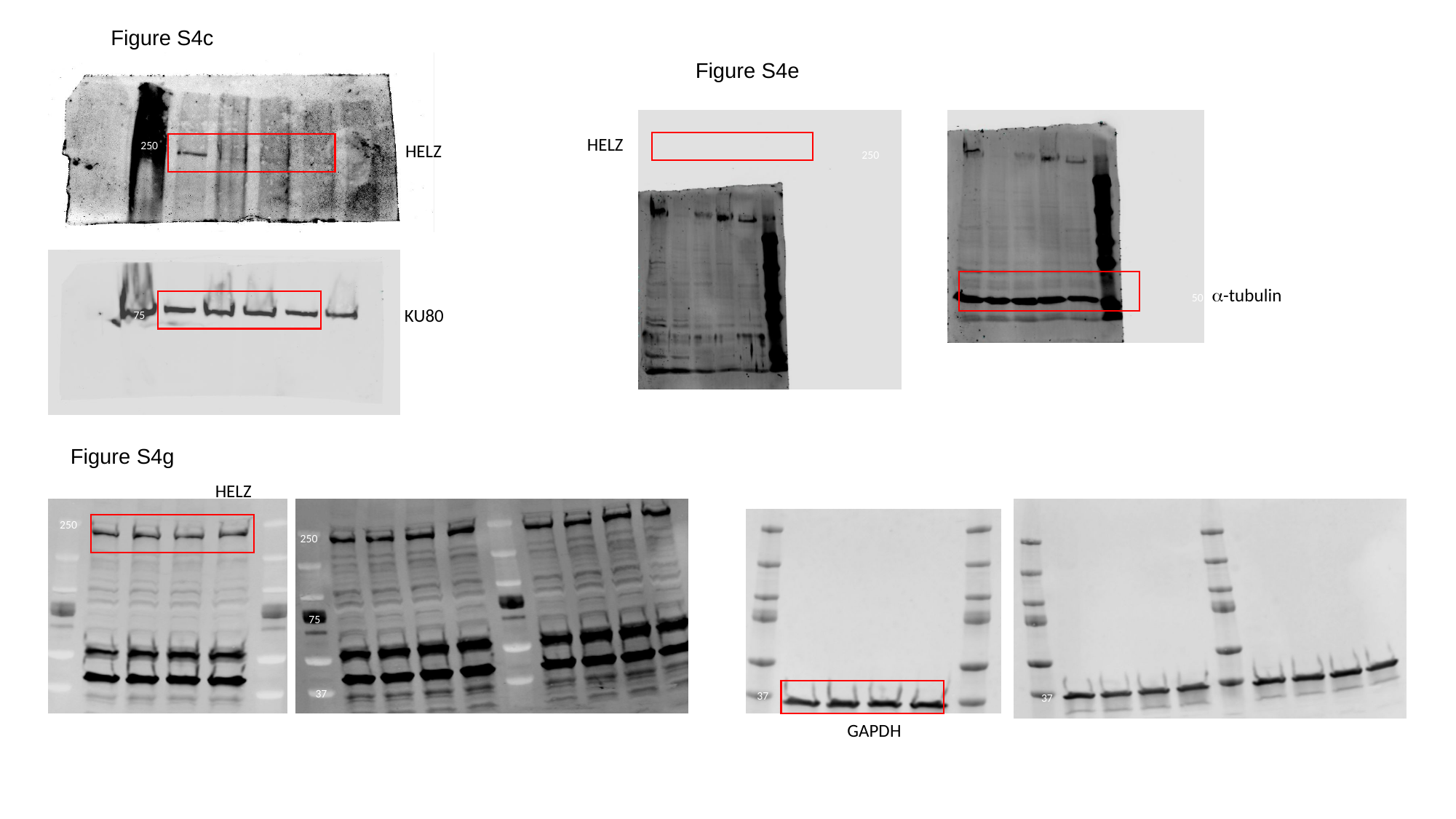

Figure S4c
Figure S4e
HELZ
250
HELZ
250
a-tubulin
50
KU80
75
Figure S4g
HELZ
250
250
75
37
37
37
GAPDH

## Slide 11
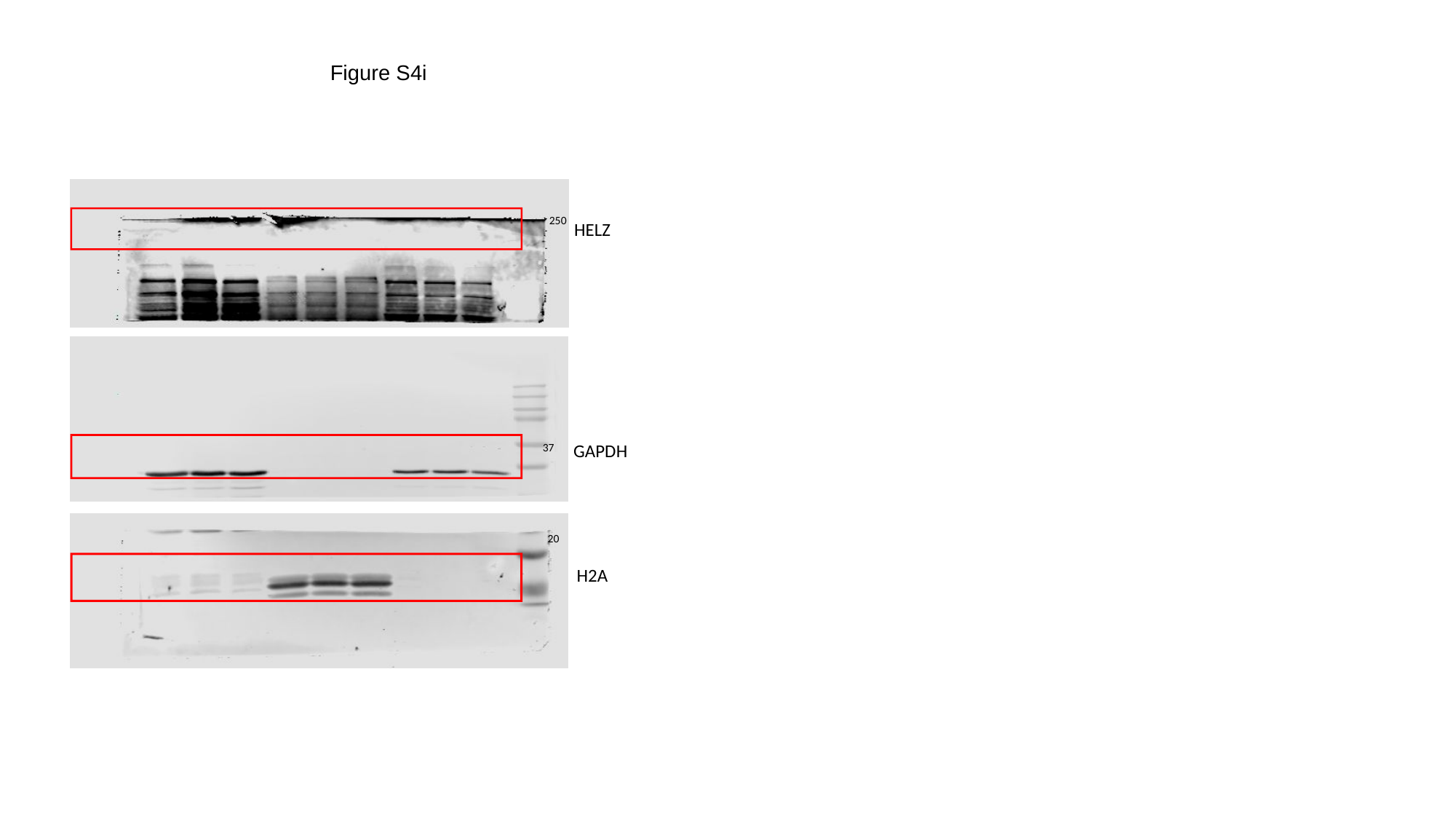

Figure S4i
250
HELZ
GAPDH
37
20
H2A

## Slide 12
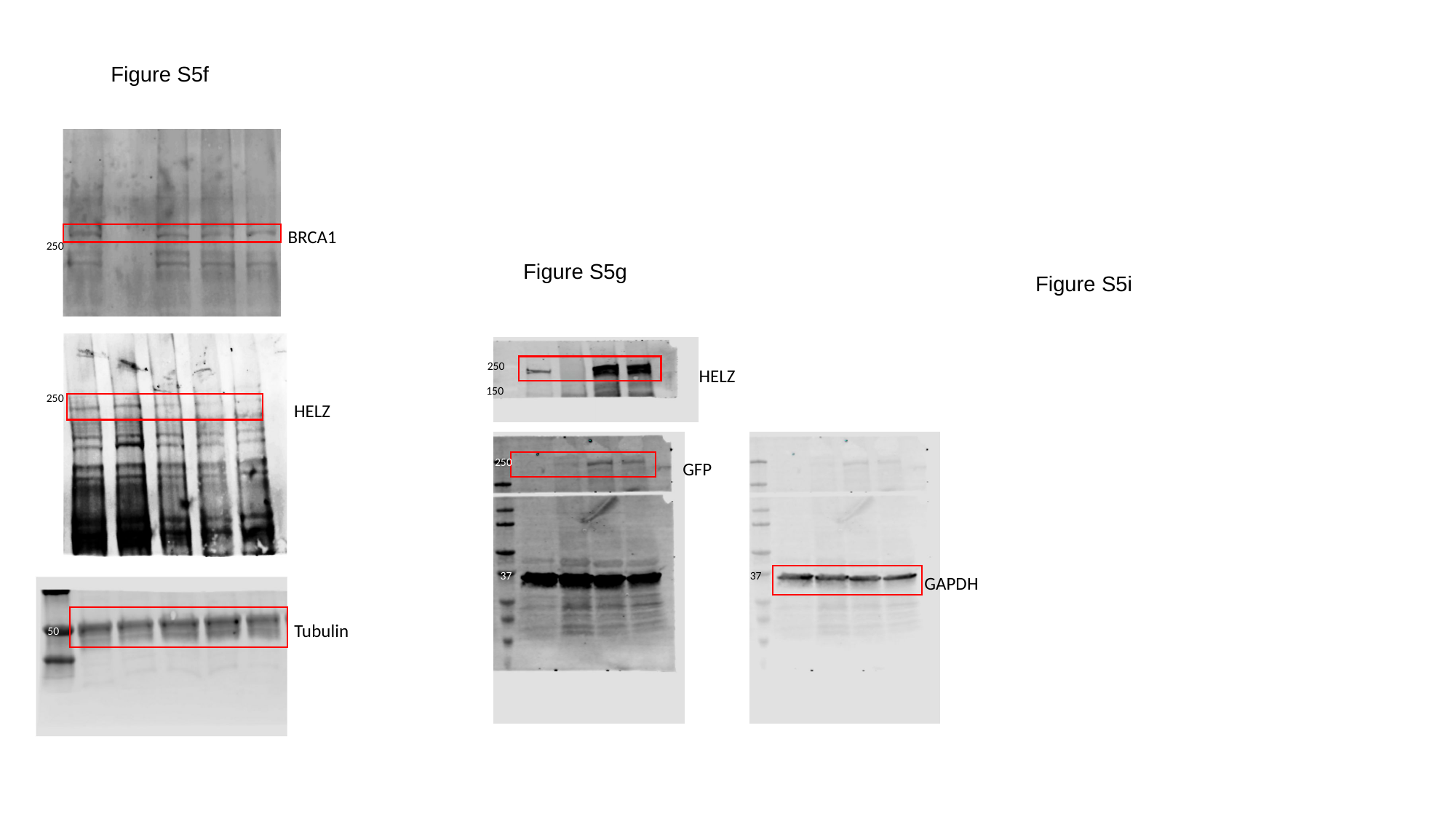

Figure S5f
BRCA1
250
Figure S5g
Figure S5i
250
HELZ
150
250
HELZ
250
GFP
37
37
GAPDH
Tubulin
50

## Slide 13
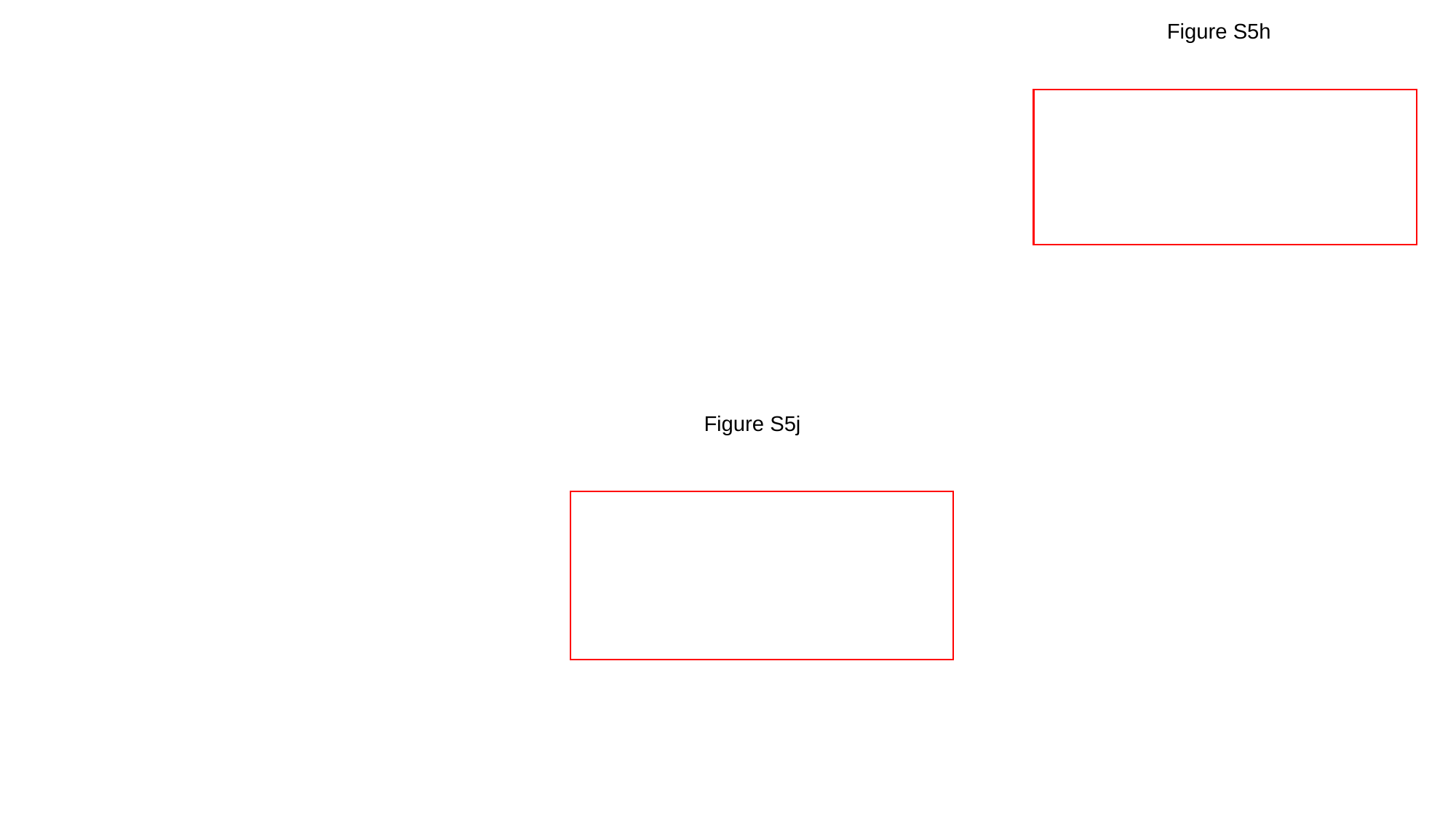

Figure S5h
Figure S5j

## Slide 14
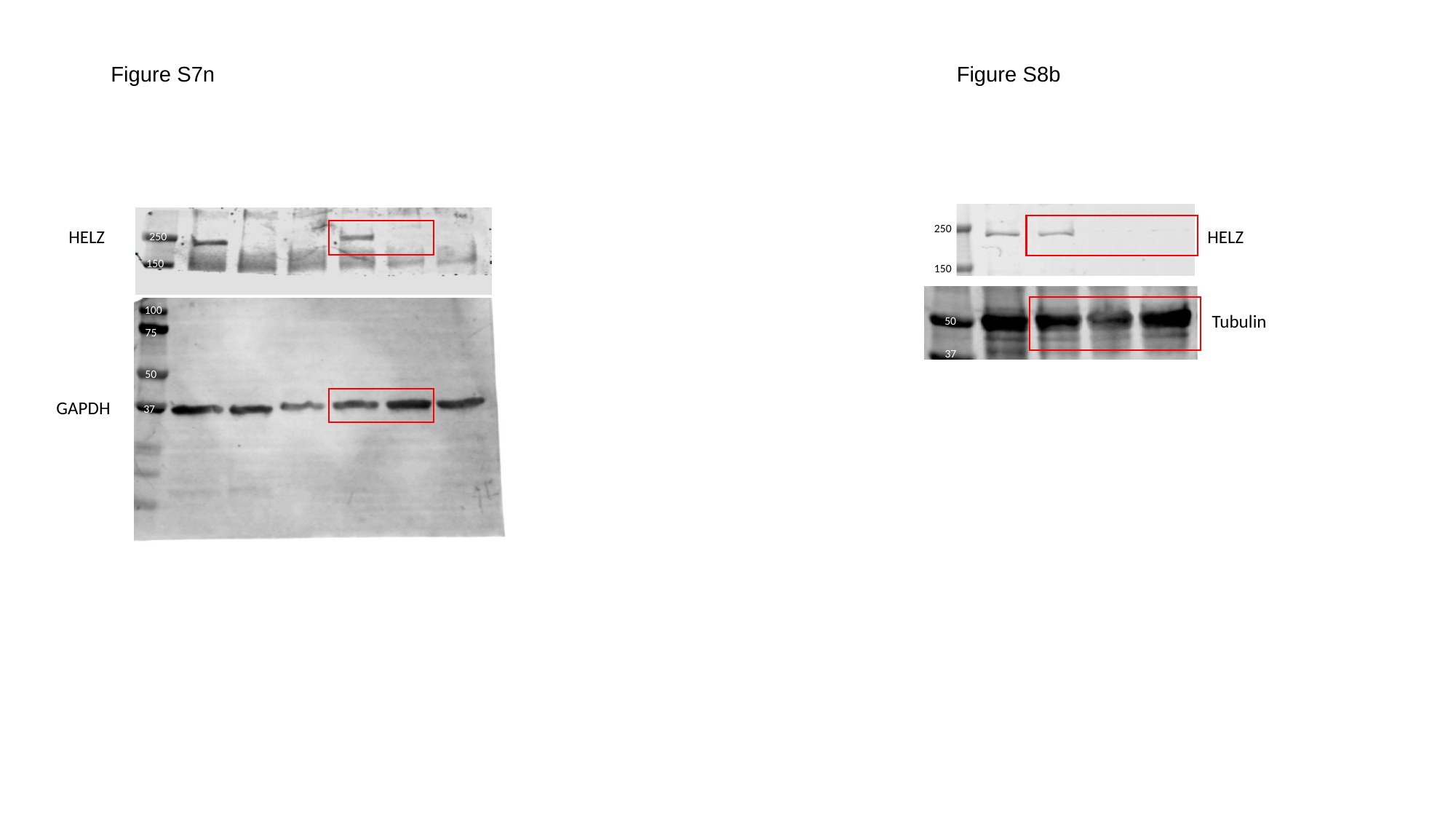

Figure S7n
Figure S8b
250
HELZ
HELZ
250
150
150
100
Tubulin
50
75
37
50
GAPDH
37

## Slide 15
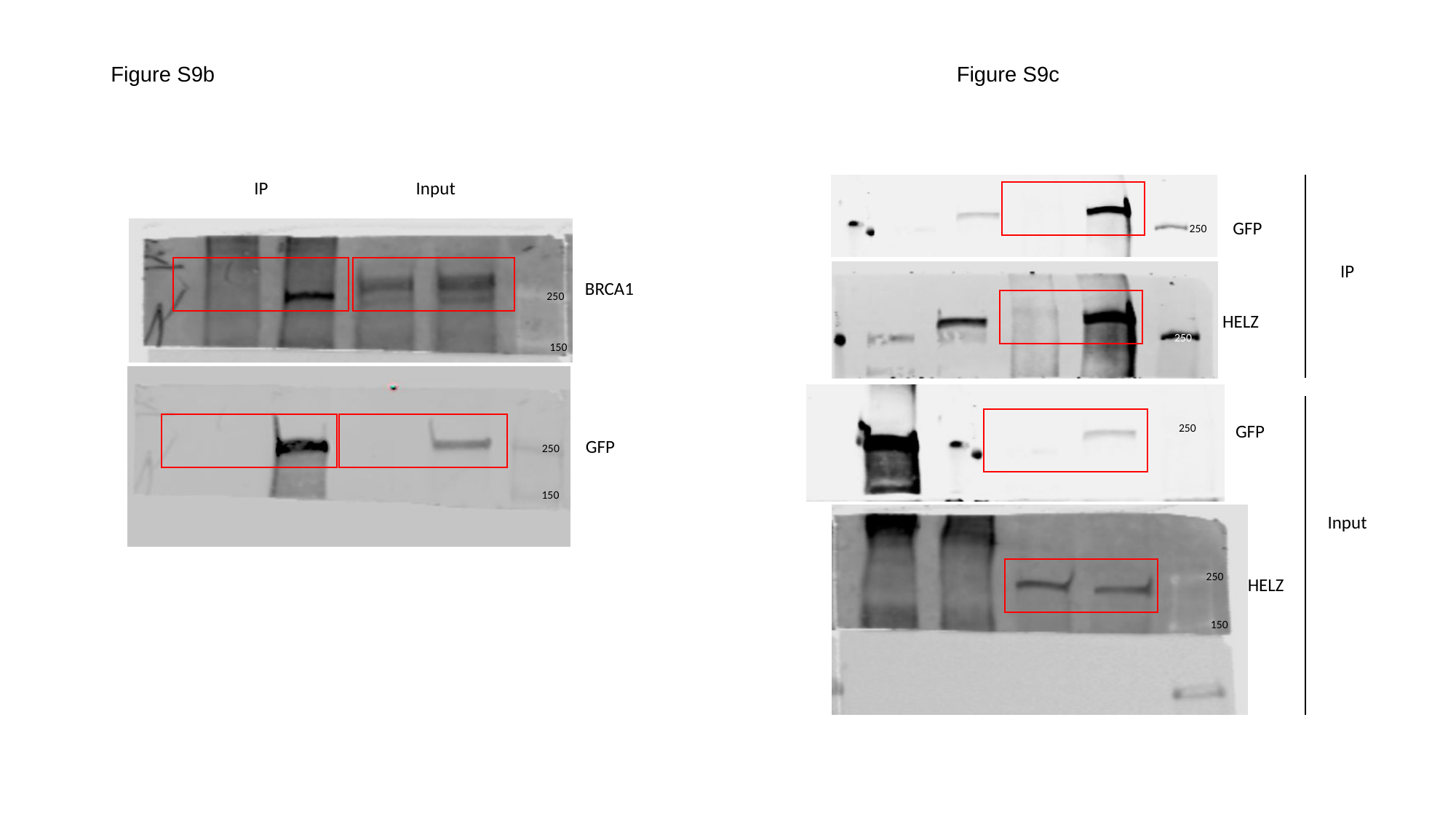

Figure S9b
Figure S9c
IP
Input
GFP
250
IP
BRCA1
250
HELZ
250
150
GFP
250
GFP
250
150
Input
250
HELZ
150

## Slide 16
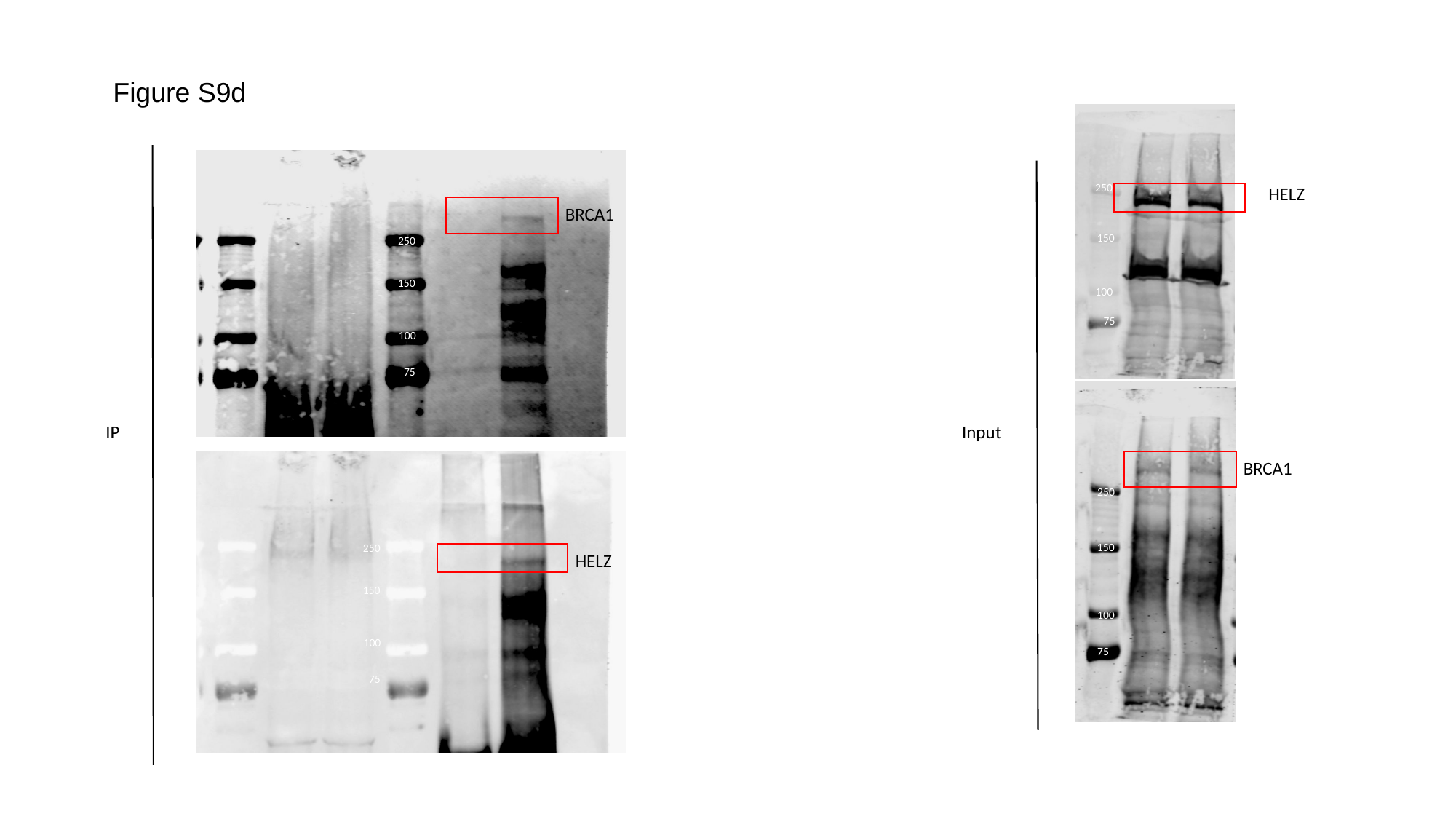

Figure S9d
250
HELZ
BRCA1
150
250
150
100
75
100
75
IP
Input
BRCA1
250
150
250
HELZ
150
100
100
75
75

## Slide 17
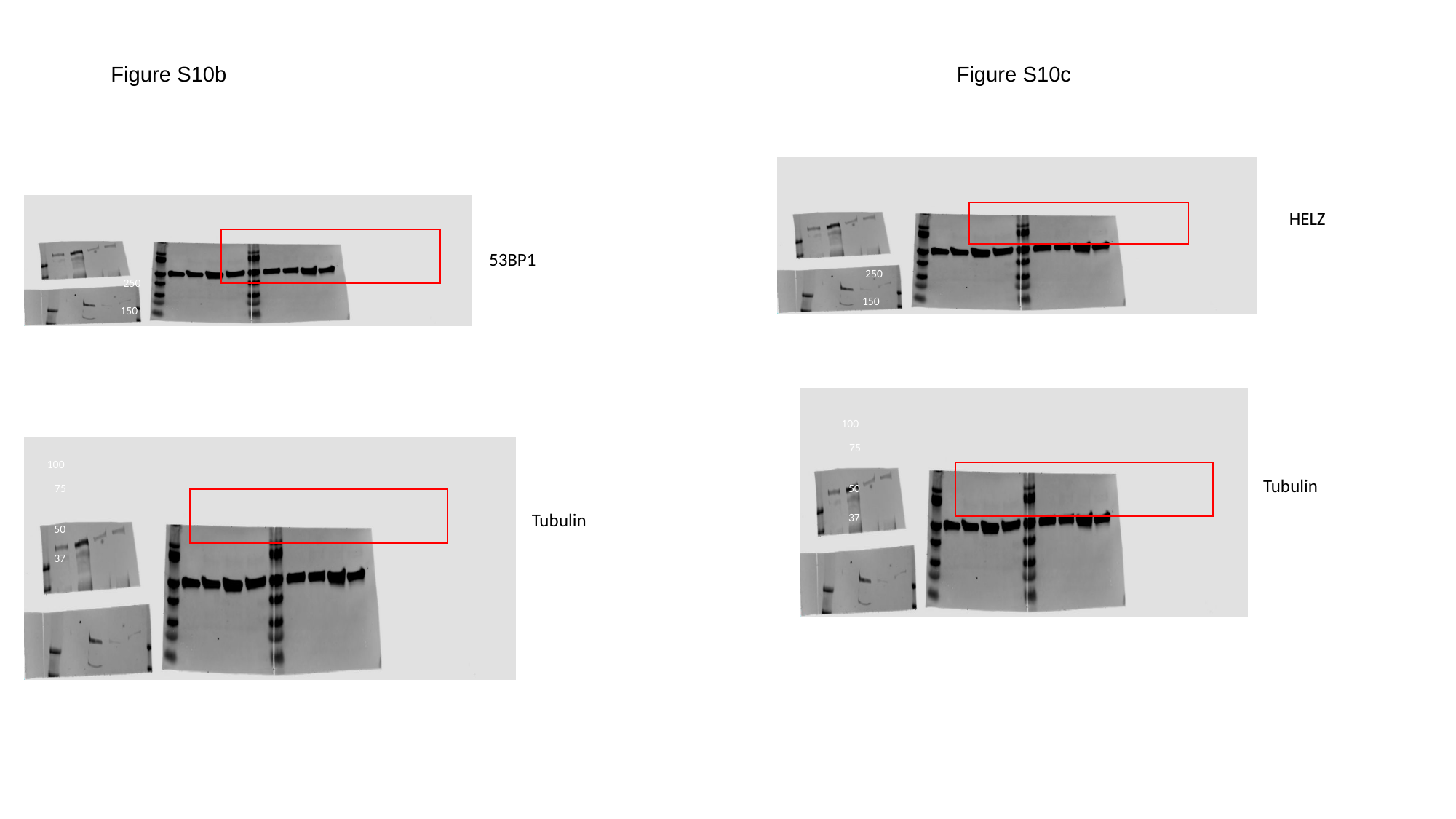

Figure S10b
Figure S10c
HELZ
53BP1
250
250
150
150
100
75
100
Tubulin
75
50
Tubulin
37
50
37
