# Supplementary material for: HELZ is a RNA-DNA helicase that resolves R loops to facilitate homologous recombination repair
Source: Nat Commun. 2026 Jul 23;17:6968. doi: 10.1038/s41467-026-75089-3 (PMC13396810; doi:10.1038/s41467-026-75089-3)
Supplement: Supplementary file 6 — Transparent Peer Review file [file 41467_2026_75089_MOESM6_ESM.pdf]

nature portfolio

Peer Review File

HELZ is a RNA-DNA helicase that resolves R loops to facilitate homologous recombination repair

Corresponding Author: Dr David Yu

This file contains all reviewer reports in order by version, followed by all author rebuttals in order by version.

Version 0:

Reviewer comments:

Reviewer #1

(Remarks to the Author)
In the manuscript "HELZ is a RNA-DNA helicase that resolves R loops to facilitate homologous recombination repair", the authors perform an RNAi screen to identify genes involved in etoposide resistance and identify HELZ as a potential candidate. They present evidence that HELZ functions as an RNA-DNA helicase, resolving R-loops and promoting homologous recombination (HR) repair. The study further suggests that HELZ supports BRCA1 recruitment to DNA double-strand breaks, thereby facilitating DNA resection and HR. Overall, the work is straightforward and includes several well-executed experiments, and the manuscript is written in a generally clear manner. However, some of the claims—particularly the proposed interaction between HELZ and BRCA1—are not sufficiently supported, and several points need to be clarified before the manuscript is suitable for publication in Nature Communications.

1. Colony formation assay methods (Fig. 1c–d, Fig. S4d): The description of the colony formation assays is incomplete. In addition to U2OS, two other cell lines were used, but these are not mentioned in the Methods. It is also unclear whether cells were treated with etoposide and SG continuously for 10–14 days. Furthermore, the rationale for seeding fewer cells at low doses of damage and more cells at high doses is not explained. Typically, equal numbers of cells should be seeded regardless of the level of genotoxic stress.

2. Inconsistent terminology (R loop vs. R-loop): Throughout most of the manuscript, the authors use the term R loop. However, in the second paragraph of page 9 and in several figure legends (e.g., Fig. S6), the term R-loop is used instead.

3. Fig. S7g–j: It is unclear why the percentage of 53BP1-positive cells was quantified for U2OS cells (panels g–h), whereas the number of 53BP1 foci was quantified for HeLa cells (panels i–j). The authors should justify this difference in analysis. In addition, if RPE cells were examined, the data is suggested be shown rather than noted as "data not shown."

4. Fig. 3f–g: The figure legend states that U2OS cells were depleted of HELZ for 72h, treated with 4 Gy IR, then treated with RNase H or RNase A for 1h before immunofluorescence analysis. However, it is unclear how the RNases were introduced into live cells. If the cells were permeabilized for RNase treatment, it is difficult to reconcile how they would still be able to undergo DNA resection and Rad51 foci formation. This requires clarification.

5. Fig. 3a, f, i: Several DAPI-stained images show intense and diffuse blue signals outside of the nucleus. These do not resemble micronuclei but rather appear to be mycoplasma contamination. The authors should verify this.

6. Fig. 4b: The PLA images lack appropriate negative controls. A single staining of HELZ or BRCA1 should be included, or alternatively, siHELZ or siBRCA1 treatment should be used to confirm that the signal reflects the proximity of the two proteins. In addition, the DAPI images in the bottom panel appear oversaturated and should be adjusted.

7. Co-IP conditions: From the Methods and figure legends, it appears that no DNase or benzonase was used during co-immunoprecipitation. Without these nucleases, the risk of false-positive interactions mediated by nucleic acids cannot be excluded.

8. Page 12, lines 12–14: The sentence "Given that HELZ depletion impairs HR by …" is misleading, as it implies that HELZ is required for R-loop formation or accumulation. The phrasing should be clarified to avoid this interpretation.

9. RNase treatments: The concentrations of RNase H and RNase A used in multiple experiments are not specified in the text.

10. Fig. 4f: Under siHELZ conditions, BRCA1 is expected to be unbound from chromatin and may not even remain in the nucleus (as suggested by the ~50% reduction shown in Fig. 4f–g). It is therefore unclear how the addition of RNases would restore BRCA1 association with chromatin if BRCA1 is no longer present in the nucleoplasm (noting the nearly 100% double-positive cells in Fig. 4g).

11. Resazurin primarily measures metabolic activity, which does not necessarily reflect true cell viability. It is suggested that the Y-axis should be relabeled to “OD value” or “relative ratio” rather than “viability.”

12. Fig. 1 legend (line 10): The legend states “HELZ depletion causes hypersensitivity to etoposide (d) and IR (d). As etoposide is shown in panel c, the legend should be corrected.

13. Fig. S4 legend: The sentence “Mean and SD from three independent replicas is shown” is duplicated. In addition, panel S4f requires quantification of the HELZ blot, and panel S4g lacks input samples, which are necessary for proper interpretation.

14. Methods (page 10, line 2): The text states “before fixation with RNase A”. Does it mean fixation with PFA instead?

Reviewer #2

(Remarks to the Author)
Review – Haji-Seyed-Javadi et al. 2025

In this manuscript, Haji-Seyed-Javadi and colleagues identify HELZ, a helicase previously described for its role in R-loop resolution, as an important gene involved in double-strand break (DSB) repair. In clonogenic and microscopy-based assays, HELZ depletion leads to hypersensitivity to DNA-damaging agents and an increased number of genomic instability markers (γH2AX, 53BP1 nuclear bodies, micronuclei). Chromatin immunoprecipitation (ChIP)-qPCR experiments reveal that R-loops specifically accumulate at sites of DNA breaks typically repaired by homologous recombination (HR), while DRIP-seq shows a global increase in R-loops upon HELZ depletion. Consistent with HELZ promoting HR at DSBs, DNA reporter assays demonstrate a drastic reduction in HR following HELZ depletion. At DNA break sites, HELZ depletion prevents the accumulation of BRCA1, single-stranded DNA (ssDNA), and RAD51. Finally, co-immunoprecipitation (co-IP) experiments suggest a potential interaction between BRCA1 and HELZ. Notably, most phenotypes are rescued by ectopic expression of HELZ or RNaseH1, an endonuclease that specifically degrades R-loops. The authors also present convincing in vitro data showing that the ATPase activity of HELZ is important for its function in HR.

Many other helicases have been reported to impact DNA damage signaling and repair due to their ability to degrade R-loops. The findings of this study are novel given the substrate specificity of HELZ, which differs from previously described helicases that remove R-loops.

The experiments presented support a role for HELZ in promoting DNA repair via HR. However, the current version of the manuscript lacks important controls to further support the authors' conclusions.

Major Issues:

As mentioned, DRIP-seq experiments reveal a genome-wide increase in R-loops in HELZ-depleted cells (Figure S6), suggesting that HELZ’s function is not specific to DSBs. The ChIP-qPCR on DivA cells (Figure 1k-i) was performed only at HR-prone sites. Can the authors rule out that this phenotype does not occur at sites prone to non-homologous end joining (NHEJ) in this system? DRIP-seq results also suggest that HELZ is broadly distributed across the genome and not specifically localized to DSBs. An analysis of the chromatin-bound fraction of HELZ with or without treatment (rather than cytoplasmic vs nuclear localization) would help clarify this point.

Based on the findings, the authors conclude that HELZ depletion prevents BRCA1 recruitment to DSB sites, impairing resection and HR repair. Further evidence is needed to support this model. If HELZ’s function is specific to BRCA1 recruitment, then depletion of 53BP1 in HELZ-depleted cells should rescue HR and RAD51 recruitment to damage sites. Additionally, while the authors investigated 53BP1 body formation, they should confirm that HELZ depletion does not affect 53BP1 recruitment to damage sites. Using DNA repair assays that monitor NHEJ would also reinforce the specificity of HELZ’s role in HR.

The interaction between HELZ and BRCA1 has not previously been observed via IP or proximity labeling assays (BioGRID). Can the authors rule out that this interaction is chromatin-independent by including benzonase treatment prior to IP in their protocol?

The authors claim that HELZ is important at DSBs; however, fork reversal rather than DSBs is induced upon treatment with low concentrations of camptothecin. The rationale for using different DNA-damaging agents and the types of damage they induce should be clearly explained throughout the manuscript.

In the BrdU assays (Figure 3J), a cell cycle assessment using antibodies against PCNA or Cyclin A should be included to confirm that the signal is specific to S-phase. A negative control, such as siRNA against CtIP, should also be used.

The main novelty of the manuscript lies in HELZ's specific binding to ssDNA and its ability to unwind RNA-DNA hybrids. Comparing HELZ's activity to another helicase, such as Senataxin, in vitro would strengthen this finding.

Minor Issues:

Figure S8C: The rationale for using siCHK1 as a control and the percentage of cells in each cell cycle phase should be provided.

Page 5, line 16: Correct the spelling of "RNF8."

Page 11, line 22: The phrase "physical interaction" should be removed, as it can only be used if the interaction is demonstrated via pulldown assays with recombinant proteins.

Some citations are outdated (close to 10 years old). Given the rapid evolution of the field, the authors should consider updating them with more recent references.

Reviewer #3

(Remarks to the Author)
In this manuscript, Haji-Seyed-Javadi et al demonstrates that HELZ is a RNA-DNA helicase that works on hybrids with a 5' RNA overhang. This activity helps resolve R loops at DSB sites to facilitate BRCA1 recruitment and thus DSB end resection for efficient homologous recombination repair. This new role of HELZ explains its involvement in maintenance of genome stability and resistance to DSB-inducing agents. This is a novel and interesting study. The authors have also shown a lot of evidence to support their claims. The major concern is about data organization and manuscript writing. There are also some concerns about experimental controls. This reviewer supports its publication if the authors can adequately address the following concerns:

Major concerns:

Page 6 lines 5-12: Starting from sacituzumab……, better wording/grammar is badly needed for this part. It is very confusing.

Page 7 line 20: I think HELZ prevents accumulation of R loops in cells dependently of its ATPase activity thus far. Helicase activity isn't demonstrated here. Page 8 lines 8-12: This is connected to page 7 line 20. I think one of the claims should be that the ATPase-dead K674N mutant is also dead in RNA-DNA hybrid unwinding.

Page 8 lines 6-12: A SDS-PAGE gel image of the purified WT and K674N HELZ can be helpful. In addition, a negative EMSA control using e.g. ssDNA should help enhance the claim about binding and a negative unwinding control using e.g. a 5'ssDNA overhang should enhance the claim about unwinding (Fig. 2f-2g).

Minor concerns:

Etoposide as a topoisomerase II inhibitor and a chemotherapy drug should be mentioned in the introduction or early in the first paragraph of result description. It would support the rationale for the etoposide sensitivity screening.

Page 5 lines 1-2: This sentence is repetitive to what Fig. 1a legend describes.

Page 5 line 17: Fig. 1b should be indicated for this claim.

Page 7 lines 11-12: I think the authors are trying to say HELZ prevents accumulation of endogenous R loops and RNaseH treatment validates (I would not use rescue here) the results.

Page 7 line 16: I believe the authors are talking about Fig. S4g, but not S5g.

Page 7 lines 13-16: It seems that HELZ is playing an essential role in preventing etoposide-induced R loops based on the results.

Page 8 line 4: Fig. 2e.

Page 8 lines 23-24 to Page 9 line 1: Does this mean that siHELZ causes more R-loop peaks?

Page 9 lines 8-22: for formality, many R-loops in this paragraph should be replaced by "R loops".

Page 26 line 24 Fig. 1 legends: It is DIvA.

Version 1:

Reviewer comments:

Reviewer #1

(Remarks to the Author)
The revised manuscript includes significant new results; not only has the functional role of HELZ been further clarified, but the overall study is now better supported by the new data. Additionally, the authors have adequately addressed most of my earlier concerns. However, several points still require further clarification:

1. In the new Fig. 4e, Benzonase and RNase H treatments were included to rule out the possibility that the HELZ-BRCA1 association is nucleic acid-dependent. However, the quality of this blot, particularly the BRCA1 signal, is inferior to that in Fig. 4d. Furthermore, while Supplementary Figs. 9d and 9e demonstrate that ectopically expressed HELZ pulls down endogenous BRCA1 (and vice versa), the association between the two endogenous proteins was not tested. Additionally, a control using a GFP-only vector was missing in both Fig. 4d-e and Supplementary Fig. 9d-e to exclude non-specific binding.

2. The authors identified HELZ through an initial siRNA screen in etoposide-resistant lung cancer cell line H128, which is a compelling starting point. However, the subsequent studies on were primarily conducted in U2OS and breast cancer cell lines. Any special rationale or explanation for this transition between model systems?

3. In the initial screen for etoposide-resistance genes, siATR was included as a positive control. Interestingly, in the supplementary table for the primary screen, another ATR entry (Gene ID 545) yielded results opposite to the siATR control; this discrepancy should be addressed. Moreover, since the HELZ-BRCA1 interaction is proposed to be critical for R-loop resolution and HR repair, it is unclear why BRCA1 itself was not identified in the initial screen. The authors should discuss these points in the Discussion section.

4. In the legend for Fig. S4, line 8, "rescue in MDA-MB-231 cells from (d)." Should this be BT-549 cells, as panel (d) appears to feature the BT-549 cells?

5. In the legend for Fig. S8, line 8, "depletion of HELZ and CHK1 for 72 h." I think CHK1 result was removed.

6. In the legend for Fig. S8, line 8, "U2OS cells were transfected with HELZ, CtIP or NT siRNA." However, CtIP is not shown in panels e and f; instead, BRCA1 is presented. Please clarify.

7. The siRNA sequences for 53BP1 and ATRIP were not listed in the Methods section.

8. Throughout the manuscript, there are inconsistencies in the observed MW of HELZ. In several blots, the band appears at approximately 250 kDa, while in others, it is located significantly below the 250 kDa.

9. Page 20, line 3, “GFP antibody (1 mg per 2 mg lysate for IP)”: Did the authors mean 1 μg antibody for IP?

Reviewer #2

(Remarks to the Author)
The authors nicely addressed all the points raised.

Reviewer #3

(Remarks to the Author)
The authors have adequately addressed all my concerns, particularly my major one with sufficient new data. I support the publication of this manuscript without further questions.

Version 2:

Reviewer comments:

Reviewer #1

(Remarks to the Author)
I am satisfied with the responses and support publication. My only remaining suggestion is for the authors to standardize the cell line names. For instance, "U2OS-265", "U20S-265", and "U20S-235" are used interchangeably for the same "mCherry-LacI-Fok1 U2OS cell." As all scientific issues are resolved, I waive any further review.

Open Access This Peer Review File is licensed under a Creative Commons Attribution 4.0 International License, which permits use, sharing, adaptation, distribution and reproduction in any medium or format, as long as you give appropriate credit to the original author(s) and the source, provide a link to the Creative Commons license, and indicate if changes were made.
In cases where reviewers are anonymous, credit should be given to 'Anonymous Referee' and the source.
The images or other third party material in this Peer Review File are included in the article's Creative Commons license, unless indicated otherwise in a credit line to the material. If material is not included in the article's Creative Commons license and your intended use is not permitted by statutory regulation or exceeds the permitted use, you will need to obtain permission directly from the copyright holder.
To view a copy of this license, visit https://creativecommons.org/licenses/by/4.0/

Reviewer 1, General Comments:

In the manuscript "HELZ is a RNA-DNA helicase that resolves R loops to facilitate homologous recombination repair", the authors perform an RNAi screen to identify genes involved in etoposide resistance and identify HELZ as a potential candidate. They present evidence that HELZ functions as an RNA-DNA helicase, resolving R-loops and promoting homologous recombination (HR) repair. The study further suggests that HELZ supports BRCA1 recruitment to DNA double-strand breaks, thereby facilitating DNA resection and HR. Overall, the work is straightforward and includes several well-executed experiments, and the manuscript is written in a generally clear manner. However, some of the claims—particularly the proposed interaction between HELZ and BRCA1—are not sufficiently supported, and several points need to be clarified before the manuscript is suitable for publication in Nature Communications.

We greatly appreciate Reviewer #1 in taking the time to review our manuscript and provide the thoughtful comments and suggestions.

Reviewer 1, Specific Comments:

Major Points:

1. Colony formation assay methods (Fig. 1c–d, Fig. S4d): The description of the colony formation assays is incomplete. In addition to U2OS, two other cell lines were used, but these are not mentioned in the Methods. It is also unclear whether cells were treated with etoposide and SG continuously for 10–14 days. Furthermore, the rationale for seeding fewer cells at low doses of damage and more cells at high doses is not explained. Typically, equal numbers of cells should be seeded regardless of the level of genotoxic stress.

Thank you for bringing this oversight to our attention. We have now clarified in the text of the Methods and Figure Legends that U2OS, MDA-MB-231, and BT-549 cells were treated continuously for 10-14 days with etoposide or SG and that different cell numbers were seeded initially and subsequently normalized to optimize colony number for counting for the colony formation assays.

2. Inconsistent terminology (R loop vs. R-loop): Throughout most of the manuscript, the authors use the term R loop. However, in the second paragraph of page 9 and in several figure legends (e.g., Fig. S6), the term R-loop is used instead.

As well-suggested, these inconsistencies have now been corrected with the use of "R loop" throughout the manuscript.

3. Fig. S7g–j: It is unclear why the percentage of 53BP1-positive cells was quantified for U2OS cells (panels g–h), whereas the number of 53BP1 foci was quantified for HeLa cells (panels i–j). The authors should justify this difference in analysis. In addition, if RPE cells were examined, the data is suggested be shown rather than noted as "data not shown."

We agree with the Reviewer that we should have been more consistent in presenting this data. As requested, percent 53BP1 positive cells for both U2OS and HeLa cells are now presented in new Supplementary Fig. S7i and k. In addition, increased spontaneous γH2AX following HELZ depletion in h-TERT immortalized RPE-1 cells are now presented in new Supplementary Fig. S7c-d, which support our original interpretation of the data that HELZ depletion leads to genomic instability.

4. Fig. 3f–g: The figure legend states that U2OS cells were depleted of HELZ for 72h, treated with 4 Gy IR, then treated with RNase H or RNase A for 1h before immunofluorescence analysis. However, it is unclear how the RNases were introduced into live cells. If the cells were permeabilized for RNase treatment, it is difficult to reconcile how they would still be able to undergo DNA resection and Rad51 foci formation. This requires clarification.

We should have been clearer. We have now clarified in the text of the Methods for new Fig. 3f-g and 4f-g that cells were treated with RNase H or RNase A for 1 h following permeabilization in live cells before fixation as adapted from Gritti et al., *EMBO J*, 2022, PMID 36215697, where RNase H treatment after permeabilization and before fixation in live cells also rescued impairment in IR-induced BRCA1 foci following DIS3 depletion. We also include new data validating under more physiologic conditions that mCherry-RNase H1 overexpression in U2OS cells alleviates the impairment in IR-induced RAD51foci from HELZ depletion (new Supplementary Fig. S8e-f). Combined with our data showing that RNase H or RNase A treatment or GFP-RNase H1 overexpression in U2OS cells alleviates the impairment in BRCA1 localization to DSBs from HELZ depletion (new Fig. 4f-i) and mCherry-RNase H1 WT but not catalytically inactive D10R/E48R overexpression alleviates the impairment in IR-induced BrdU exposure following HELZ depletion (new Fig. 3i and Supplementary Fig. S8k), these data strongly support our model that HELZ promotes DNA end resection and HR by preventing R loop accumulation.

5. Fig. 3a, f, i: Several DAPI-stained images show intense and diffuse blue signals outside of the nucleus. These do not resemble micronuclei but rather appear to be mycoplasma contamination. The authors should verify this.

Improved quality images are provided for several of the panels in new Fig. 3a, f, and i. We validated that our cell lines are mycoplasma free and additionally experimentally reproduced these experiments with mycoplasma free cell lines, including in new Supplementary Fig. S8k, but still observed some diffuse blue signal which may be due to cellular debris from transfection.

6. Fig. 4b: The PLA images lack appropriate negative controls. A single staining of HELZ or BRCA1 should be included, or alternatively, siHELZ or siBRCA1 treatment should be used to confirm that the signal reflects the proximity of the two proteins. In addition, the DAPI images in the bottom panel appear oversaturated and should be adjusted.

We agree with the Reviewer in regards to these points. We experimentally reproduced the PLA experiments using HELZ and BRCA1 alone controls and found an increase in PLA of HELZ and BRCA1 following etoposide treatment (new Supplementary S9b-c), supporting our original

interpretation of the data of an increase in HELZ and BRCA1 close proximity following etoposide treatment. Improved DAPI images are also now provided in new Fig. 4b.

7. Co-IP conditions: From the Methods and figure legends, it appears that no DNase or benzonase was used during co-immunoprecipitation. Without these nucleases, the risk of false-positive interactions mediated by nucleic acids cannot be excluded.

We completed the well-suggested experiment and found that the co-IP of HELZ-Myc-GFP and endogenous BRCA1 is preserved following benzonase nuclease or RNase H treatment (new Fig. 4e), implying that the interaction of HELZ and BRCA1 is not nucleic acid mediated.

8. Page 12, lines 12–14: The sentence "Given that HELZ depletion impairs HR by …" is misleading, as it implies that HELZ is required for R-loop formation or accumulation. The phrasing should be clarified to avoid this interpretation.

As well-suggested, we have now revised the text in the Results section to now read "Given that HELZ depletion impairs HR in a RNA-mediated manner…"

9. RNase treatments: The concentrations of RNase H and RNase A used in multiple experiments are not specified in the text.

We should have been clearer and have now provided the concentrations for RNase H and A for different experiments in the text of the Methods section.

10. Fig. 4f: Under siHELZ conditions, BRCA1 is expected to be unbound from chromatin and may not even remain in the nucleus (as suggested by the ~50% reduction shown in Fig. 4f–g). It is therefore unclear how the addition of RNases would restore BRCA1 association with chromatin if BRCA1 is no longer present in the nucleoplasm (noting the nearly 100% double-positive cells in Fig. 4g).

We should have been clearer. Our data show that HELZ depletion in cells impairs BRCA1 localization to both Fok1 and IR-induced DSBs and that treatment with RNase H/A or GFP-RNase H1 overexpression alleviates the impairment in BRCA1 localization to DSBs from HELZ depletion (new Fig. 4f-i and Supplementary Fig. S9f-i), suggesting that HELZ promotes BRCA1 recruitment to DSBs by preventing accumulation of R loops. As BRCA1 localization to DSBs is not fully impaired with HELZ depletion and retention of BRCA1 in the nucleus is still observed which is more apparent in the IR treated cells (new Fig. 4h and Supplementary S9h), it is possible that there is sufficient nucleoplasmic BRCA1 localization for restoration of BRCA1 localization to DSBs with HELZ depletion. As restoration of BRCA1 localization to DSBs is observed with treatment with RNase H/A or GFP-RNase H1 overexpression, we remain confident in our original interpretation of the data that HELZ facilitates BRCA1 recruitment to DSBs by preventing accumulation of R loops.

11. Resazurin primarily measures metabolic activity, which does not necessarily reflect true cell viability. It is suggested that the Y-axis should be relabeled to "OD value" or "relative ratio"

rather than "viability."

We agree with the Reviewer. As well-suggested, the Y-axis for new Supplementary Fig. S4a-b using resazurin has now been relabeled "Relative Ratio" with the figure legend indicating measurement of metabolic activity.

12. Fig. 1 legend (line 10): The legend states "HELZ depletion causes hypersensitivity to etoposide (d) and IR (d). As etoposide is shown in panel c, the legend should be corrected.

Thank you for bringing this oversight to our attention. We have now revised the text for the figure legend for new Fig. 1.

13. Fig. S4 legend: The sentence "Mean and SD from three independent replicas is shown" is duplicated. In addition, panel S4f requires quantification of the HELZ blot, and panel S4g lacks input samples, which are necessary for proper interpretation.

Thank you for bringing this oversight to our attention. We have now revised the duplicate sentence from the Figure Legend for Supplementary Fig. S4. Quantification of the blot in new Supplementary S4g from 3 independent replicas is now shown in new Supplementary Fig. S4h. Input samples are now presented in new Supplementary Fig. S4i.

14. Methods (page 10, line 2): The text states "before fixation with RNase A". Does it mean fixation with PFA instead?

Thank you for this comment. This oversight has now been corrected replacing "RNase A" with "PFA."

Reviewer 2, General Comments:

In this manuscript, Haji-Seyed-Javadi and colleagues identify HELZ, a helicase previously described for its role in R-loop resolution, as an important gene involved in double-strand break (DSB) repair. In clonogenic and microscopy-based assays, HELZ depletion leads to hypersensitivity to DNA-damaging agents and an increased number of genomic instability markers (γH2AX, 53BP1 nuclear bodies, micronuclei). Chromatin immunoprecipitation (ChIP)-qPCR experiments reveal that R-loops specifically accumulate at sites of DNA breaks typically repaired by homologous recombination (HR), while DRIP-seq shows a global increase in R-loops upon HELZ depletion. Consistent with HELZ promoting HR at DSBs, DNA reporter assays demonstrate a drastic reduction in HR following HELZ depletion. At DNA break sites, HELZ depletion prevents the accumulation of BRCA1, single-stranded DNA (ssDNA), and RAD51. Finally, co-immunoprecipitation (co-IP) experiments suggest a potential interaction between BRCA1 and HELZ. Notably, most phenotypes are rescued by ectopic expression of HELZ or RNaseH1, an endonuclease that specifically degrades R-loops. The authors also present convincing in vitro data showing that the ATPase activity of HELZ is important for its function in HR.

Many other helicases have been reported to impact DNA damage signaling and repair due to

their ability to degrade R-loops. The findings of this study are novel given the substrate specificity of HELZ, which differs from previously described helicases that remove R-loops.

The experiments presented support a role for HELZ in promoting DNA repair via HR. However, the current version of the manuscript lacks important controls to further support the authors' conclusions.

We greatly appreciate Reviewer #2 in taking the time to review our manuscript and provide the thoughtful comments and suggestions.

Reviewer 2, Specific Comments:

Major Points:

1. As mentioned, DRIP-seq experiments reveal a genome-wide increase in R-loops in HELZ-depleted cells (Figure S6), suggesting that HELZ's function is not specific to DSBs. The ChIP-qPCR on DivA cells (Figure 1k-i) was performed only at HR-prone sites. Can the authors rule out that this phenotype does not occur at sites prone to non-homologous end joining (NHEJ) in this system? DRIP-seq results also suggest that HELZ is broadly distributed across the genome and not specifically localized to DSBs. An analysis of the chromatin-bound fraction of HELZ with or without treatment (rather than cytoplasmic vs nuclear localization) would help clarify this point.

These are excellent points. As well-suggested, we performed chromatin IP (ChIP) with quantitative PCR (qPCR) using U2OS DIvA (DSB Inducible via AsiSI) cells and also found enriched accumulation of HELZ at NHEJ-prone DSBs (new Supplementary Fig. S4f) in addition to our originally presented data indicating enriched accumulation of HELZ at HR-prone DSBs (new Fig. 1k-l), supporting our original interpretation of the data that HELZ binds to DSBs and suggesting that this is not specific to HR-prone vs NHEJ-prone DSBs. We also performed biochemical fractionation of HELZ with or without etoposide treatment and found a mild increase in chromatin bound HELZ following etoposide treatment (new Supplementary Fig. S4i), consistent with our observation that HELZ binds to DNA following induction of DSBs.

2. Based on the findings, the authors conclude that HELZ depletion prevents BRCA1 recruitment to DSB sites, impairing resection and HR repair. Further evidence is needed to support this model. If HELZ's function is specific to BRCA1 recruitment, then depletion of 53BP1 in HELZ-depleted cells should rescue HR and RAD51 recruitment to damage sites. Additionally, while the authors investigated 53BP1 body formation, they should confirm that HELZ depletion does not affect 53BP1 recruitment to damage sites. Using DNA repair assays that monitor NHEJ would also reinforce the specificity of HELZ's role in HR.

We completed the well-suggested experiment and found that 53BP1 depletion rescued the impairment in IR-induced RAD51 foci from HELZ depletion (new Supplementary Fig. S10a-d), consistent with a role for HELZ in promoting HR by facilitating BRCA1 recruitment.

In addition, using the EJ7-GFP classical-NHEJ (c-NHEJ) reporter integrated in HEK293 cells, we observed a mild decrease in c-NHEJ as well as IR-induced 53BP1 foci with HELZ depletion (new Supplementary Fig. S8c and S8g-h) suggesting that HELZ may also promote c-NHEJ.

3. The interaction between HELZ and BRCA1 has not previously been observed via IP or proximity labeling assays (BioGRID). Can the authors rule out that this interaction is chromatin-independent by including benzonase treatment prior to IP in their protocol?

This is an excellent suggestion. We completed the experiment and found that the co-IP of HELZ-Myc-GFP and endogenous BRCA1 is preserved following benzonase nuclease or RNase H treatment (new Fig. 4e), suggesting that the interaction of HELZ and BRCA1 is not nucleic acid mediated.

4. The authors claim that HELZ is important at DSBs; however, fork reversal rather than DSBs is induced upon treatment with low concentrations of camptothecin. The rationale for using different DNA-damaging agents and the types of damage they induce should be clearly explained throughout the manuscript.

Rationale for use of ionizing radiation (IR); etoposide, a topoisomerase I inhibitor; camptothecin (CPT), a topoisomerase II inhibitor; and sacituzumab govitecan (SG), an antibody drug conjugate (ADC) consisting of the active irinotecan metabolite SN-38 conjugated to anti-Trop-2 monoclonal antibody, which all induce DSBs that HELZ responds to, suggesting that HELZ responds generally to DSBs has now been provided in the text of the Results section.

5. In the BrdU assays (Figure 3J), a cell cycle assessment using antibodies against PCNA or Cyclin A should be included to confirm that the signal is specific to S-phase. A negative control, such as siRNA against CtIP, should also be used.

Controls for CtIP knockdown and PCNA co-labeling for the BrdU assay are now presented in new Supplementary Fig. S8k, which support our original interpretation of the data that HELZ depletion impairs DNA end resection. We have left the original BrdU data in new Fig. 3i-j because it contains an important γH2AX co-labeling control.

6. The main novelty of the manuscript lies in HELZ's specific binding to ssDNA and its ability to unwind RNA-DNA hybrids. Comparing HELZ's activity to another helicase, such as Senataxin, in vitro would strengthen this finding.

We performed the suggested experiment and found that recombinant SETX has slightly stronger unwinding activity for RNA/DNA hybrids with 5'ssRNA overhangs compared with recombinant HELZ and that recombinant SETX but not HELZ unwinds DNA/DNA hybrids with 5'ssDNA overhangs, consistent with a prior report that SETX unwinds both RNA/DNA hybrids with 5'ssRNA overhangs and DNA/DNA hybrids with 5'ssDNA overhangs (Hasanova et al., NAR, 2023, PMID36864660). However, due to insufficient purity of our recombinant SETX, which is highly challenging to purify, we are not fully confident in this comparison so have not included it in the manuscript but have provided it below for the Reviewer.

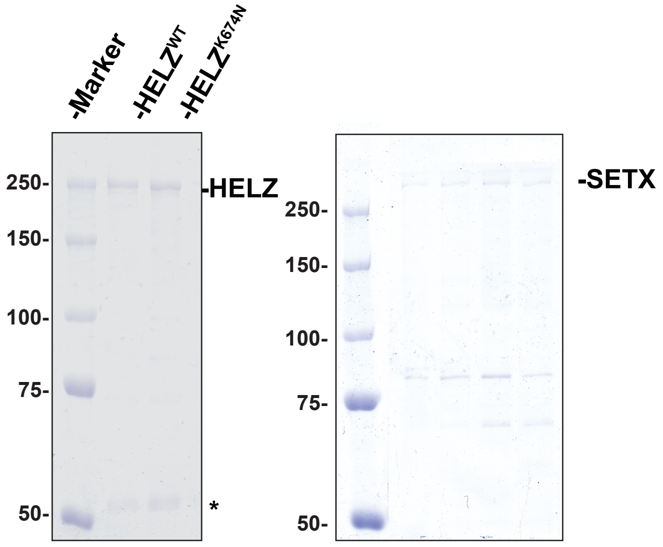

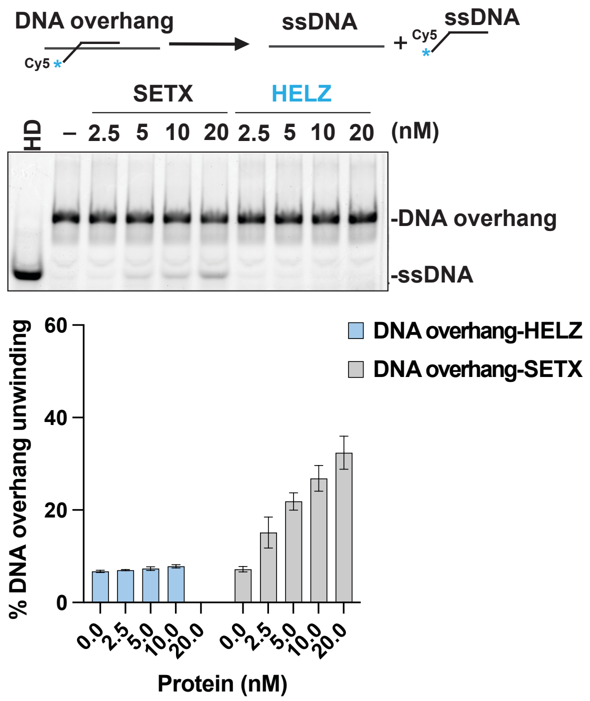

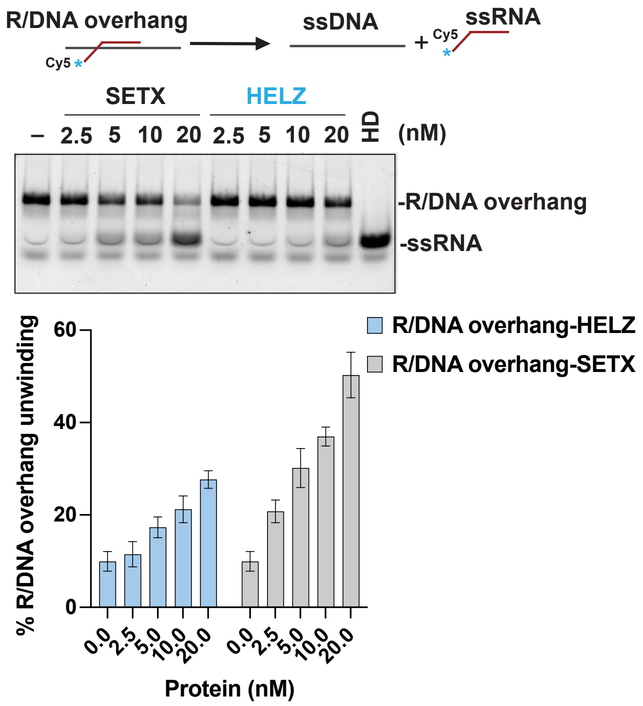

Minor Points:

1. Figure S8C: The rationale for using siCHK1 as a control and the percentage of cells in each cell cycle phase should be provided.

As requested, the percentage of cells in each cell cycle phase following HELZ depletion are now provided in new Supplementary Fig. S8d. siCHK1 was originally presented as a positive control for disrupting cell cycle distribution; however, on retrospect, we agree as implied by the

Reviewer that there may not be sufficient rationale for using it to inform on HELZ depletion in this context and thus have revised it from the figure.

2. Page 5, line 16: Correct the spelling of "RNF8."

Thank you for bringing this oversight to our attention which has now been corrected.

3. Page 11, line 22: The phrase "physical interaction" should be removed, as it can only be used if the interaction is demonstrated via pulldown assays with recombinant proteins.

We agree with the Reviewer and have revised "physical" from the text.

4. Some citations are outdated (close to 10 years old). Given the rapid evolution of the field, the authors should consider updating them with more recent references.

Updated references are now provided as requested.

Reviewer 3, General Comments:

In this manuscript, Haji-Seyed-Javadi et al demonstrates that HELZ is a RNA-DNA helicase that works on hybrids with a 5' RNA overhang. This activity helps resolve R loops at DSB sites to facilitate BRCA1 recruitment and thus DSB end resection for efficient homologous recombination repair. This new role of HELZ explains its involvement in maintenance of genome stability and resistance to DSB-inducing agents. This is a novel and interesting study. The authors have also shown a lot of evidence to support their claims. The major concern is about data organization and manuscript writing. There are also some concerns about experimental controls. This reviewer supports its publication if the authors can adequately address the following concerns:

We greatly thank the reviewer for taking the time to review our manuscript and provide the thoughtful comments and suggestions.

Reviewer 3, Specific Comments:

Major Points:

1. Page 6 lines 5-12: Starting from sacituzumab……, better wording/grammar is badly needed for this part. It is very confusing.

We should have been clearer and have now revised the text in the Results section for better readability of the description of SG and rescue of SG hypersensitivity with RNase H1.

2. Page 7 line 20: I think HELZ prevents accumulation of R loops in cells dependently of its ATPase activity thus far. Helicase activity isn't demonstrated here. Page 8 lines 8-12: This is connected to page 7 line 20. I think one of the claims should be that the ATPase-dead K674N

mutant is also dead in RNA-DNA hybrid unwinding.

We agree with the Reviewer in regards to these points and thus have revised the text in the Results section to indicate that "HELZ prevents accumulation of R loops in cells through its ATPase activity" and later that "ATPase dead K674N is also dead for RNA-DNA unwinding."

3. Page 8 lines 6-12: A SDS-PAGE gel image of the purified WT and K674N HELZ can be helpful. In addition, a negative EMSA control using e.g. ssDNA should help enhance the claim about binding and a negative unwinding control using e.g. a 5'ssDNA overhang should enhance the claim about unwinding (Fig. 2f-2g).

These are all excellent suggestions. An SDS-PAGE gel of insect purified recombinant HELZ WT and K674N is now presented in new Supplementary Fig. S5l. We also found no evidence that recombinant HELZ binds to ssDNA using EMSA (new Supplementary Fig. S5j-k) to complement our nucleic acid cellular pulldown assays showing specificity of HELZ binding to ssRNA and RNA/DNA hybids with ssRNA overhangs but not ssDNA or pure RNA/DNA hybrids (new Fig 2e). In addition, we performed unwinding assays of DNA/DNA hybrids with ssDNA overhangs with insect purified HELZ and SETX and found that SETX but not HELZ unwinds DNA/DNA hybrids with ssDNA overhangs. Due to insufficient purity of our recombinant SETX, which is highly challenging to purify, we are not fully confident in this data so have not included it in the manuscript but have provided it below for the Reviewer.

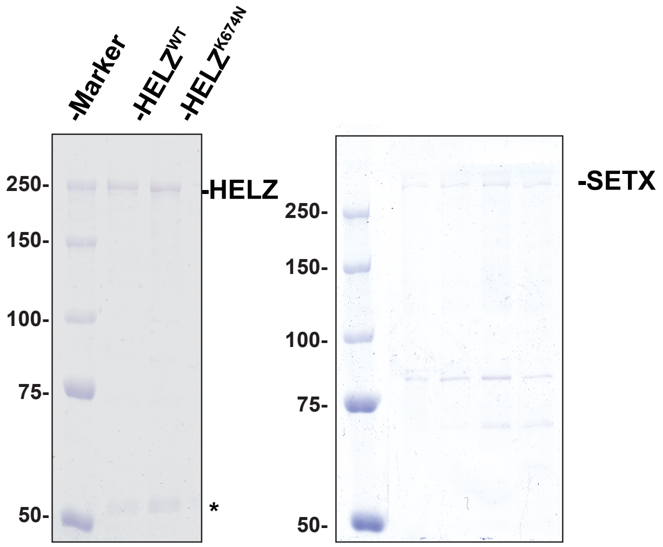

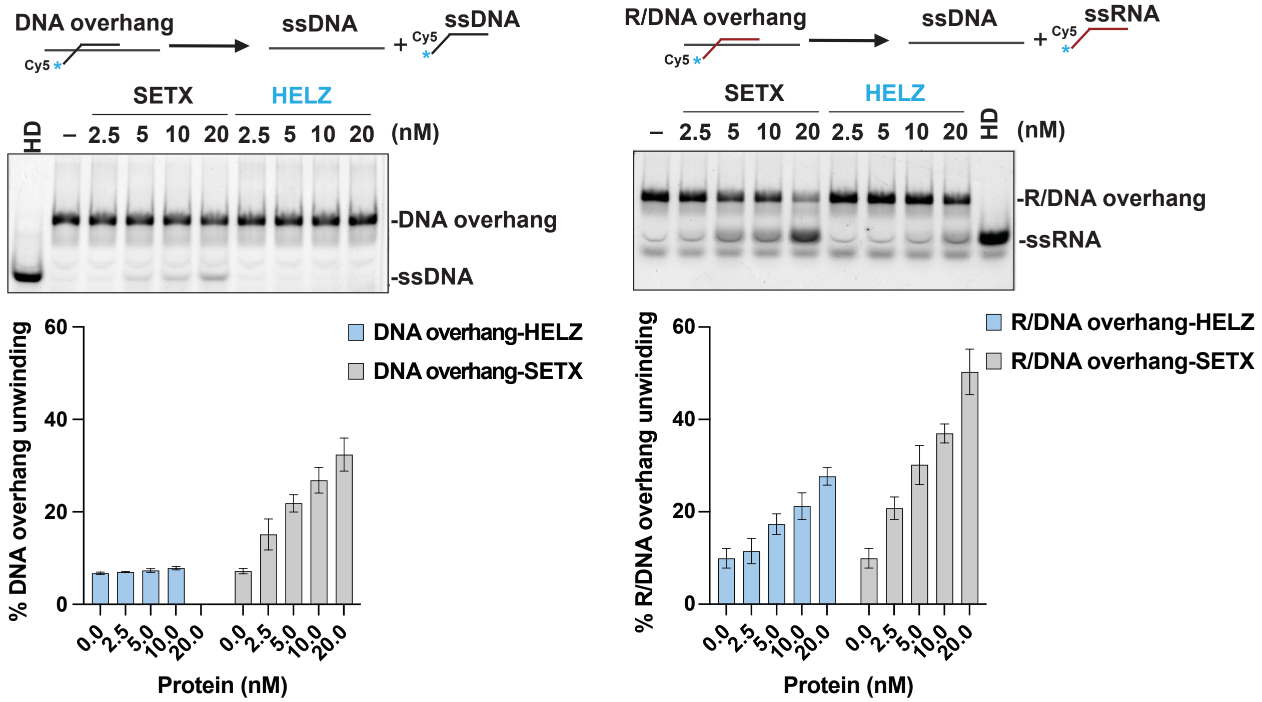

Minor Points:

1. Etoposide as a topoisomerase II inhibitor and a chemotherapy drug should be mentioned in the introduction or early in the first paragraph of result description. It would support the rationale for the etoposide sensitivity screening.

Excellent suggestion. The text of the Results section has been revised with specific language describing etoposide as a topoisomerase II inhibitor and chemotherapeutic drug.

2. Page 5 lines 1-2: This sentence is repetitive to what Fig. 1a legend describes.

As well-suggested, we have revised the text of the Results section to remove overlap with new Fig. 1a legend.

3. Page 5 line 17: Fig. 1b should be indicated for this claim.

This is now done.

4. Page 7 lines 11-12: I think the authors are trying to say HELZ prevents accumulation of endogenous R loops and RNaseH treatment validates (I would not use rescue here) the results.

We have now replaced "validated" with "alleviated."

5. Page 7 line 16: I believe the authors are talking about Fig. S4g, but not S5g.

We should have been clearer. New Supplementary Fig. S5g refers to the western blot validating HELZ knockdown and overexpression of GFP-HELZ WT and K674N for the slot blot analysis in new Fig. 2c.

6. Page 7 lines 13-16: It seems that HELZ is playing an essential role in preventing etoposide-induced R loops based on the results.

We agree with the Reviewer's interpretation which is described in the Results section.

7. Page 8 line 4: Fig. 2e.

Thank you for pointing out this oversight which has now been corrected.

8. Page 8 lines 23-24 to Page 9 line 1: Does this mean that siHELZ causes more R-loop peaks?

This is correct. Our data do suggest that HELZ knockdown leads to an increase in R-loop peaks.

9. Page 9 lines 8-22: for formality, many R-loops in this paragraph should be replaced by "R loops".

As suggested, "R-loops" have now been revised to "R loops" throughout the manuscript for consistency.

10. Page 26 line 24 Fig. 1 legends: It is DIvA.

Thank you for pointing out this oversight which has now been corrected.

Reviewer 1, General Comments:

The revised manuscript includes significant new results; not only has the functional role of HELZ been further clarified, but the overall study is now better supported by the new data. Additionally, the authors have adequately addressed most of my earlier concerns. However, several points still require further clarification:

We greatly appreciate Reviewer #1 in taking the time to review our manuscript and provide the additional thoughtful comments and suggestions.

Reviewer 1, Specific Comments:

Major Points:

1. In the new Fig. 4e, Benzonase and RNase H treatments were included to rule out the possibility that the HELZ-BRCA1 association is nucleic acid-dependent. However, the quality of this blot, particularly the BRCA1 signal, is inferior to that in Fig. 4d. Furthermore, while Supplementary Figs. 9d and 9e demonstrate that ectopically expressed HELZ pulls down endogenous BRCA1 (and vice versa), the association between the two endogenous proteins was not tested. Additionally, a control using a GFP-only vector was missing in both Fig. 4d-e and Supplementary Fig. 9d-e to exclude non-specific binding.

We agree with the Reviewer in regards to these points. As suggested, we experimentally reproduced the co-IP study, including with a GFP only control and found that co-IP of HELZ-Myc-GFP but not GFP alone expressed in HEK293T cells pulls down endogenous BRCA1, which was preserved after benzonase or RNase treatment (new Fig. 4e), supporting our original interpretation of the data that the interaction of HELZ and BRCA1 is not nucleic acid mediated. We also performed co-IP of endogenous BRCA1 in HEK293T cells and found that endogenous BRCA1 pulls down endogenous HELZ (new Supplementary Fig. S9d), suggesting that endogenous BRCA1 and HELZ complex with each other.

2. The authors identified HELZ through an initial siRNA screen in etoposide-resistant lung cancer cell line H128, which is a compelling starting point. However, the subsequent studies on were primarily conducted in U2OS and breast cancer cell lines. Any special rationale or explanation for this transition between model systems?

We should have been clearer. We initially performed the etoposide sensitivity screen in etoposide-resistant H128 SCLC cell lines since etoposide is clinically used to treat SCLC; however, we found that HELZ depletion also causes hypersensitivity to DSB-inducing agents etoposide, IR, CPT, and SG in multiple other cell types, including U2OS, MDA-MB-231, and BT-549 cells, suggesting that the phenotype is not cell type specific. Subsequent mechanistic studies were performed in U2OS and other cell types commonly used to study the DNA damage response so that results can be compared to published studies.

3. In the initial screen for etoposide-resistance genes, siATR was included as a positive control. Interestingly, in the supplementary table for the primary screen, another ATR entry (Gene ID

545) yielded results opposite to the siATR control; this discrepancy should be addressed. Moreover, since the HELZ-BRCA1 interaction is proposed to be critical for R-loop resolution and HR repair, it is unclear why BRCA1 itself was not identified in the initial screen. The authors should discuss these points in the Discussion section.

This is an excellent point, which is now addressed in the Discussion section. While our primary etoposide sensitivity siRNA screen did enrich for expected DNA damage response genes, it is possible that some of the siRNA in the screen may not have led to sufficient knockdown and/or had off-target effects, which could help explain why our screen results were not saturating.

4. In the legend for Fig. S4, line 8, "rescue in MDA-MB-231 cells from (d)." Should this be BT-549 cells, as panel (d) appears to feature the BT-549 cells?

Thank you for bringing this oversight to our attention. We have clarified that the figure legend for new Supplementary Fig. S4e should refer to BT-549 cells.

5. In the legend for Fig. S8, line 8, "depletion of HELZ and CHK1 for 72 h." I think CHK1 result was removed.

Thank you for bringing this oversight to our attention. We have now revised CHK1 from the Figure Legend for new Supplementary Fig. S8d.

6. In the legend for Fig. S8, line 8, "U2OS cells were transfected with HELZ, CtIP or NT siRNA." However, CtIP is not shown in panels e and f; instead, BRCA1 is presented. Please clarify.

Thank you for bringing this oversight to our attention. We have now revised CtIP from the Figure Legend for new Supplementary Fig. S8e-f.

7. The siRNA sequences for 53BP1 and ATRIP were not listed in the Methods section.

Thank you for bringing this oversight to our attention. These sequences are now provided in the Methods section.

8. Throughout the manuscript, there are inconsistencies in the observed MW of HELZ. In several blots, the band appears at approximately 250 kDa, while in others, it is located significantly below the 250 kDa.

Some of the differences in the observed MW of HELZ are related to the use of different percent SDS-PAGE gels for different blots. Where appropriate, inconsistencies in labeling of the MW markers in relation to HELZ have now been corrected.

9. Page 20, line 3, "GFP antibody (1 mg per 2 mg lysate for IP)": Did the authors mean 1 μg antibody for IP?

This oversight has now been corrected.

Reviewer 2, General Comments:

The authors nicely addressed all the points raised.

Thank you!

Reviewer 3, General Comments:

The authors have adequately addressed all my concerns, particularly my major one with sufficient new data. I support the publication of this manuscript without further questions.

Thank you!

Response to Reviewers

Reviewer #1:

Comment: I am satisfied with the responses and support publication. My only remaining suggestion is for the authors to standardize the cell line names. For instance, "U2OS 265", "U20S-265", and "U20S-235" are used interchangeably for the same "mCherry-LacI-Fok1 U2OS cell." As all scientific issues are resolved, I waive any further review.

Response: We thank the reviewer for this helpful comment. We have carefully reviewed the entire manuscript and revised the cell line nomenclature to ensure consistency and uniform formatting throughout the text, figures, and figure legends, where applicable.
